# Net carbon emissions from African biosphere dominate pan-tropical atmospheric CO_2_ signal

**DOI:** 10.1038/s41467-019-11097-w

**Published:** 2019-08-13

**Authors:** Paul I. Palmer, Liang Feng, David Baker, Frédéric Chevallier, Hartmut Bösch, Peter Somkuti

**Affiliations:** 1 School of GeoSciences, University of Edinburgh, Edinburgh, EH9 3FF UK; 2 National Centre for Earth Observation at the University of Edinburgh, Edinburgh, EH9 3FF UK; 3 Cooperative Institute for Research in the Atmosphere, Colorado State University, Fort Collins, 80523-1375 Colorado USA; 4 Laboratoire des Sciences du Climat et de l’Environnement/IPSL, CEA-CNRS-UVSQ, Université Paris-Saclay, F-91198 Gif-sur-Yvette, France; 5 Department of Physics and Astronomy, University of Leicester, Leicester, LE1 7RH UK; 6 National Centre for Earth Observation at the University of Leicester, Leicester, LE1 7RH UK

**Keywords:** Carbon cycle, Atmospheric chemistry

## Abstract

Tropical ecosystems are large carbon stores that are vulnerable to climate change. The sparseness of ground-based measurements has precluded verification of these ecosystems being a net annual source (+ve) or sink (−ve) of atmospheric carbon. We show that two independent satellite data sets of atmospheric carbon dioxide (CO_2_), interpreted using independent models, are consistent with the land tropics being a net annual carbon emission of $({\mathrm{median}}_{{\mathrm{minimum}}}^{{\mathrm{maximum}}})$ $1.03_{ - 0.20}^{ + 1.73}$ and $1.60_{ + 1.39}^{ + 2.11}$ petagrams (PgC) in 2015 and 2016, respectively. These pan-tropical estimates reflect unexpectedly large net emissions from tropical Africa of $1.48_{ + 0.80}^{ + 1.95}$ PgC in 2015 and $1.65_{ + 1.14}^{ + 2.42}$ PgC in 2016. The largest carbon uptake is over the Congo basin, and the two loci of carbon emissions are over western Ethiopia and western tropical Africa, where there are large soil organic carbon stores and where there has been substantial land use change. These signals are present in the space-borne CO_2_ record from 2009 onwards.

## Abstract (web summary)

Tropical land ecosystems contain vast carbon reservoirs, but their influence on atmospheric CO_2_ is poorly understood. Here the authors use new carbon-observing satellites to reveal a large emission source over northern tropical Africa, where there are large soil carbon stores and substantial land use changes.

## Introduction

Tropical terrestrial ecosystems store large amounts of carbon in plants and soil, but are particularly vulnerable to changes in climate^1,2^. They release CO_2_ via autotrophic and heterotrophic respiration and via fire, and take up CO_2_ via photosynthesis. The terrestrial tropics, defined between 23.44°S and 23.44^o^N, include 30% of the global land surface and approximately a third of all Earth’s three billion trees^3^ and their stored carbon. Our knowledge of the tropical carbon budget has improved significantly over the past few decades mainly due to networks of sample plot measurements^4^, micro-meteorological measurements of carbon fluxes of forest ecosystems^5^, remote sensing of vegetation state or of land use change^6^, and sparsely-distributed ground-based mole fraction measurements^7,8^ of atmospheric CO_2_. Despite these efforts, carbon fluxes from tropical ecosystems remain one of the largest uncertainties in the global carbon cycle^9,10^ and impose a similar uncertainty on our ability to predict future climate change.

We use a range of global satellite data (“Methods”) to study the carbon cycle over the tropics from 2009 to 2017 with a focus on 2015/2016 when two independent satellites were observing atmospheric CO_2_. We use total column CO_2_ dry air mole fraction (X_CO2_) retrievals from the Japanese Greenhouse gases Observing SATellite^11^ (GOSAT) from mid-2009 until 2017 and from the NASA Orbiting Carbon Observatory^12^ (OCO-2) from late 2014 to 2017. For comparative purposes, we use an inter-calibrated network of various mole fraction data^7,8^ (“Methods”). We interpret these ground-based mole fraction and remotely-sensed column mole fraction data using three independent atmospheric transport models, driven by different a priori CO_2_ flux estimates, and their counterpart inverse methods (“Methods”). The result is a range of geographically-resolved a posteriori CO_2_ fluxes for the globe. We report our results over land as net biosphere fluxes (“Methods”), representing the net carbon flux exchange with the atmosphere from above-ground biomass and soils across sub-continental regions. To interpret these CO_2_ fluxes in terms of the underlying land surface processes, we use correlative satellite data products (Methods): vegetation indices that provide information about leaf phenology^13^; changes in water storage^14^; a measure of photosynthesis^15^; and formaldehyde columns that provide information about the location and timing of fires^16^. We use dry matter (DM) burned estimates^17^ inferred from remotely sensed land surface properties, and analysed meteorological fields of surface temperature and precipitation from the GEOS-5 (GEOS-FP) model (“Methods”).

We use three inverse methods (“Methods”) representing a range of atmospheric transport models, driving meteorology, and estimation methods. We focus on the GEOS-Chem atmospheric transport model^18^ and discuss differences with the other models. Our primary study period is 2014–2017 when there is overlap between GOSAT and OCO-2 data, coinciding with the El Niño event^19–21^. The a posteriori global atmospheric growth rate of CO_2_, inferred from ground-based data (“Methods”) and converted from satellite-based flux estimates, ranges from 4.5 to 6.1 PgC year^−1^ over our study, consistent with values inferred from the CO_2_ mass inferred directly from the atmospheric mole fraction data multiplied by the total mass of dry air in the atmosphere.

Our analysis of the GOSAT and OCO-2 data reveals that the land tropics are a net annual CO_2_ emission of $({\mathrm{median}}_{{\mathrm{minimum}}}^{{\mathrm{maximum}}})$ $1.03_{ - 0.20}^{ + 1.73}$ and $1.60_{ + 1.39}^{ + 2.11}$ petagrams (PgC) in 2015 and 2016, respectively, and larger than estimates inferred from changes in above-ground biomass^22–24^. The range of individual model estimates can be relatively large, particularly for regions where the net carbon budget is small, but nevertheless a coarse picture of the changing carbon budget emerges from our analysis. We find a robust signal over northern tropical Africa that is responsible for the majority of the pan-tropical net carbon signal, which cannot be explained by potential measurement or model biases. The largest seasonal uptake is over the northern Congo basin, as expected, and the largest emissions are found over western Ethiopia and western tropical Africa during March and April when it is hottest and driest. Although caution should be exercised when interpreting regions smaller than 1000 km, these emission focal points are a robust feature of the GOSAT record that starts in 2009. While we do not provide a definitive explanation for this seasonal signal, we argue that a comparatively small constant CO_2_ flux, e.g., from soils due to sustained land degradation^25^, could manifest as a seasonal net carbon source.

## Results

### Pan-tropical carbon flux estimates

**Fig. 1:**
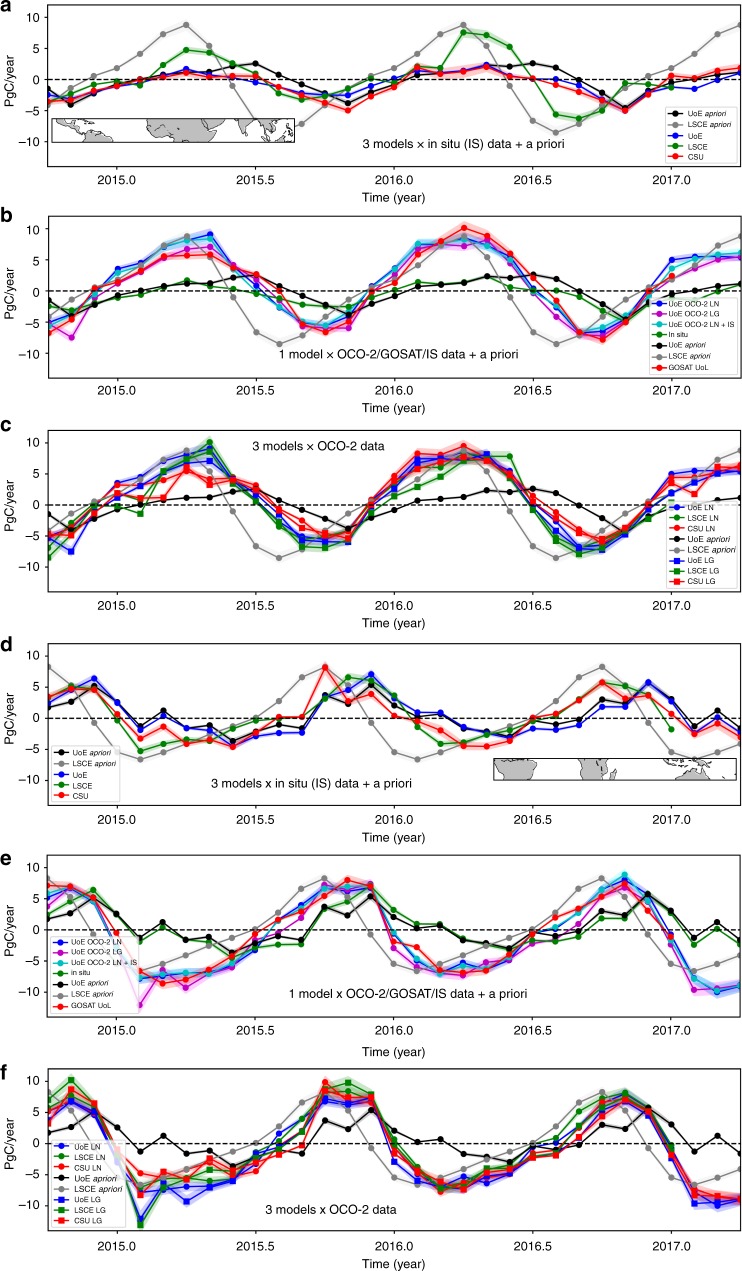
Northern and southern tropical CO_2_ fluxes. Monthly a priori and a posteriori CO_2_ fluxes (expressed as PgC year^−1^, mid-2014 to 2017) from the (**a**–**c**) Northern and (**d**–**f**) Southern tropics inferred from (**a**, **d**) in situ mole fraction measurements and from (**b**–**c**, **e**–**f**) GOSAT and OCO-2 satellite measurements of X_CO2_. Positive fluxes are from the land surface to the atmosphere. LN and LG denote X_CO2_ measurements taken using nadir and glint observing modes, respectively. The geographical regions are shown inset of each upper panel

Figures 1a and d demonstrate that the sparse ground-based measurements provide insufficient information to determine robust estimates of tropical land carbon fluxes across the three groups, even on a pan-tropical scale. Differences in atmospheric model transport, assumptions about model errors, and differences between a priori land biosphere fluxes result in sometimes-inconsistent a posteriori estimates^9,10^. This has hampered the ability of the wider Earth system science community to understand large-scale responses of the carbon cycle to climate. On a broad scale, we can make two observations. First, we find that using column observations of X_CO2_ from GOSAT and OCO-2 results in more consistent a posteriori CO_2_ flux estimates over the tropics (Fig. 1b–f), with a smaller inter-model spread of estimates^26^, and a better agreement on the phase of the seasonal cycle than using only in situ observations of CO_2_ (Fig. 1a–d). Second, the amplitude of the seasonal cycle of a posteriori CO_2_ fluxes over the northern and southern tropical lands inferred by the satellite data is generally much larger than that inferred from the in situ data (Fig. 1b–e), with the exception of LSCE that is driven by a priori fluxes from the ORCHIDEE model (“Methods”). Differences between the amplitude of the seasonal cycle inferred by GEOS-Chem using GOSAT and OCO-2 data (Fig. 1b–e) are smaller than those from different models that use the same data (Fig. 1c–f). Assumptions about data analysis therefore still play a role in the a posteriori flux estimates, but these inter-model differences are generally small compared to differences between the a posteriori and a priori flux estimates. Together, these two observations suggest that the satellite data contain substantial information about the carbon cycle. For completeness, we refer the reader to Supplementary Figs. 1–6 and Supplementary note 1 for analysis and discussion of a posteriori CO_2_ fluxes from all other regions across the world.

Table 1 shows that the land tropics are a net annual carbon emission of $1.03_{ - 0.20}^{ + 1.73}$ and $1.60_{ + 1.39}^{ + 2.11}$ PgC in 2015 and 2016, respectively, and larger than estimates inferred from changes in above-ground biomass^22–24^. We find that northern tropical fluxes are $1.54_{ - 0.12}^{ + 1.58}$PgC in 2015 that increase in 2016 to $1.72_{ + 1.61}^{ + 2.42}$PgC. Southern tropical fluxes are $- 0.26_{ - 0.55}^{ + 0.23}$PgC in 2015 and $- 0.18_{ - 0.41}^{ - 0.01}$PgC in 2016. Even on a pan-tropical scale for 2015 and 2016, reaching a consensus on the sign of the land flux (except for GOSAT ACOS data, Table 1) and on its seasonal amplitude ($1.39_{ - 0.20}^{ + 2.11}$PgC) represents a significant step forward for the carbon cycle community. For our analysis we have not quantified anomalous fluxes during El Niño^20,21^.

**Table 1:** A priori and a posteriori net biosphere CO_2_ flux estimates (PgC year^−1^, positive values are to the atmosphere) for 2015 and 2015 for individual geographical regions over the tropics inferred from GOSAT and OCO-2 X_CO2_ retrievals

– denotes absence of data. UoL and ACOS denote independent retrieval data products from the University of Leicester and the NASA Atmospheric CO_2_ Observation from Space, respectively. UoE, LSCE, and CSU denote the modelling groups from the University of Edinburgh, Laboratoire des Sciences du Climat et de l’Environnement, and Colorado State University, respectively. Colours are used to distinguish column/row groups

### Continental-scale tropical carbon flux estimates

During 2015 we find that net fluxes from tropical South America are $- 0.26_{ - 0.58}^{ + 0.04}$PgC, tropical African fluxes are$1.48_{ + 0.80}^{ + 1.95}$PgC, and from tropical Asia and tropical Australia are $- 0.13_{ - 0.45}^{ + 0.40}$PgC and $- 0.10_{ - 0.33}^{ - 0.06}$PgC, respectively (Table 1). In comparison, during 2016 tropical South America fluxes are $0.20_{ - 0.21}^{ + 0.53}$PgC, tropical African fluxes are $1.65_{ + 1.14}^{ + 2.42}$PgC, and tropical Asia and tropical Australia are $- 0.01_{ - 0.40}^{ + 0.29}$PgC and $- 0.11_{ - 0.51}^{ - 0.05}$PgC, respectively. The range of individual model estimates can be relatively large, particularly for regions where the net carbon budget is small, but nevertheless a coarse picture of the changing carbon budget emerges from our analysis (Table 1).

### Carbon flux estimates for northern tropical Africa and southern tropical South America

**Fig. 2:**
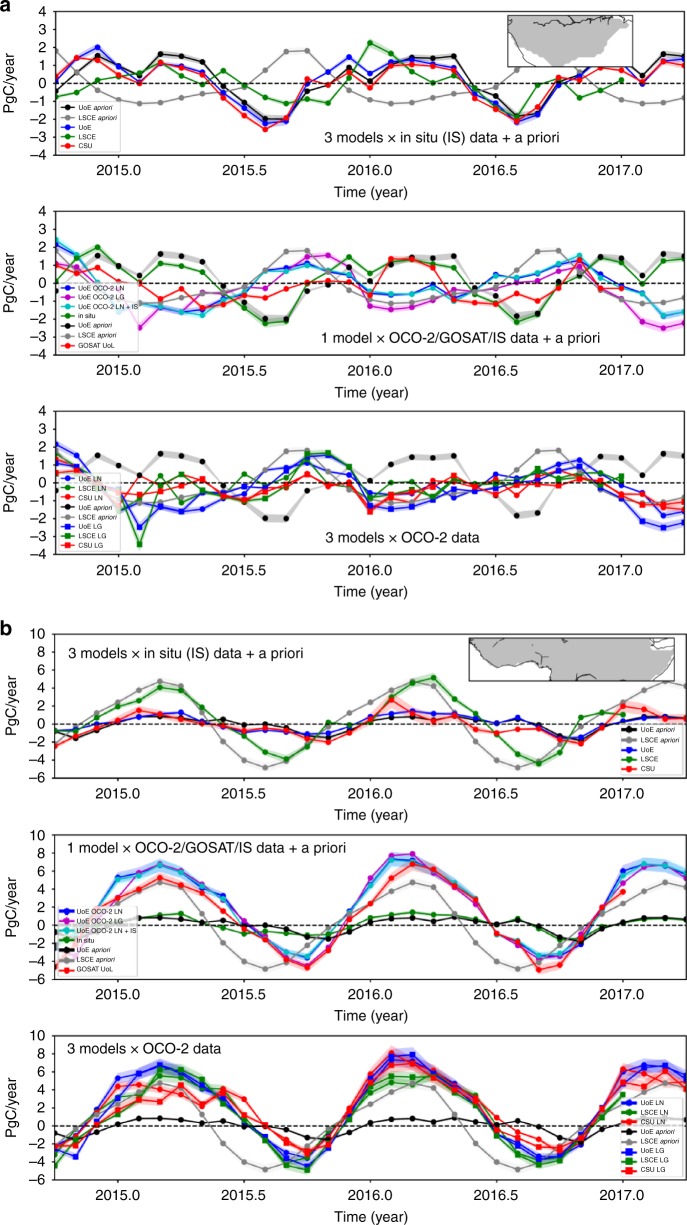
CO_2_ fluxes from tropical South America and tropical northern Africa. Monthly a priori and a posteriori CO_2_ fluxes (expressed as PgC year^−1^, mid-2014 to 2017) from **a** southern tropical South America and **b** northern tropical Africa, inferred from in situ mole fraction measurements and from GOSAT and OCO-2 satellite measurements of X_CO2_. Positive fluxes are from the land surface to the atmosphere. LN and LG denote X_CO2_ measurements taken from nadir and glint observing modes, respectively. The geographical regions are shown inset of each upper panel.

**Fig. 3:**
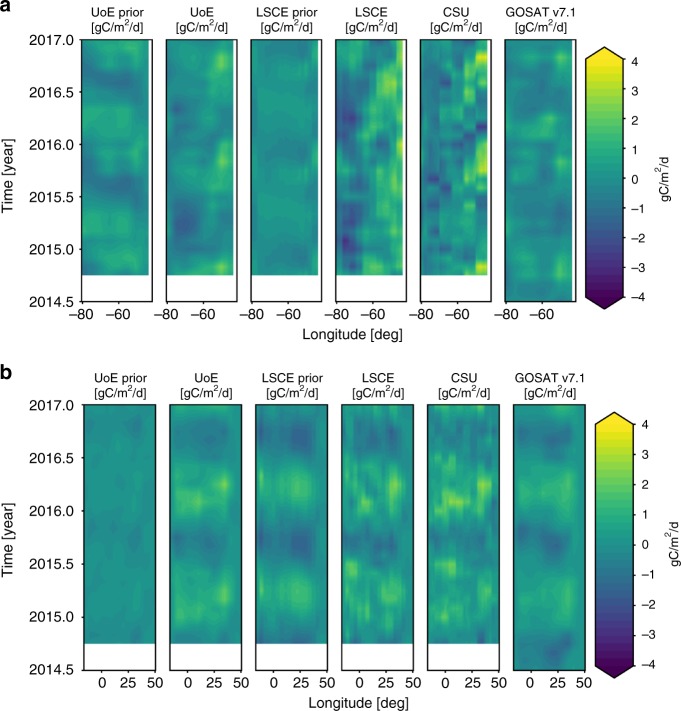
CO_2_ fluxes from tropical South America and tropical northern Africa. Hovmöller plots of monthly a priori and a posteriori CO_2_ fluxes (expressed as gC m^−2^ d^−1^, mid 2014 to 2017), averaged over the latitude domain, from **a** southern tropical South America and **b** northern tropical Africa, inferred from GOSAT and OCO-2 satellite measurements of X_CO2_ taken from the nadir observing mode. Positive fluxes are from the land surface to the atmosphere.

**Fig. 4:**
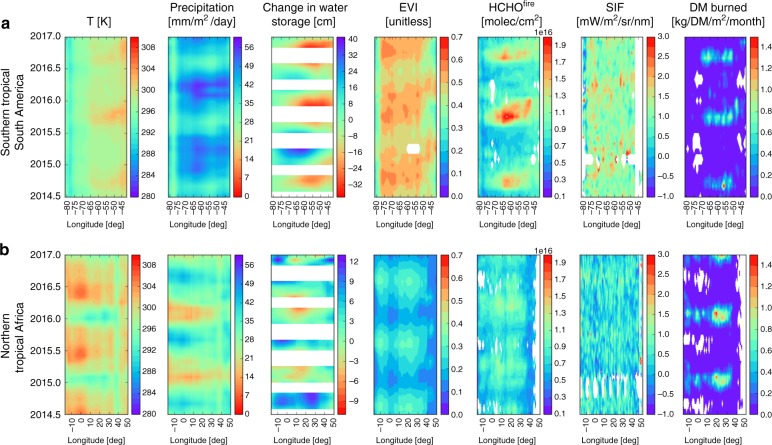
Correlative data to interpret regional X_CO2_ data. Correlative data over **a** southern tropical South America and **b** northern tropical Africa. The panels are from left to right: surface temperature (K), precipitation (mm m^−2^ day^−1^), water storage (cm), elevated vegetation index (m^2^ m^−2^), HCHO columns (molec cm^−2^) filtered for fire activity using MODIS fire counts, and dry matter (DM) burned (kg DM^−1^ m^−2^ month^−1^)

Figures 2 and 3 shows carbon budgets for two contrasting tropical regions: southern tropical South America and northern tropical Africa. To explore the ability of these satellite data to constrain fluxes on smaller spatial scales, we present our results also as latitude-mean Hovmöller plots, reflecting that physical climate variations over the tropics are typically oriented E-W. In the absence of independent CO_2_ data to evaluate these distributions, we interpret the a posteriori CO_2_ fluxes using correlative satellite observations (Fig. 4).

Over southern tropical South America (Fig. 2a), UoE a posteriori fluxes are shifted from the a priori seasonal cycle, resulting in a better agreement with fluxes inferred from the same data using different models (Fig. 2a, bottom panel). A posteriori flux estimates inferred from GOSAT lie between a priori values and the fluxes inferred from OCO-2, reflecting the superior data density of OCO-2; fluxes inferred from GOSAT are insignificantly different from a priori values during early 2016 due to a very low density of measurements during this period. Differences in the spatial and temporal CO_2_ flux distributions (Fig. 3a) demonstrate current limitations in our ability to infer spatial distributions of CO_2_ fluxes^26,27^. We find that the a posteriori distributions of carbon flux over the El Niño period resemble the E-W dipole pattern of water storage (Fig. 4a), with larger positive (negative) anomalies towards the east (west) corresponding to larger positive (negative) CO_2_ fluxes. The El Niño period also saw anomalous fire activity in the 2015 dry season (Fig. 4a) that reflects anomalous high temperatures and drought conditions, which increase the susceptibility of vegetation to ignite.

We find that GOSAT and OCO-2 X_CO2_^28^ data consistently assign the largest seasonal cycle of carbon fluxes over the tropics to northern tropical Africa (Fig. 2b and 3b) with that region being responsible for the unexpectedly large pan-tropical net source of carbon (Table 1, Fig. 1). Over this region, we find close agreement between the a posteriori flux estimates on small spatial and temporal scales (Fig. 3b). The largest seasonal uptake is over the northern Congo basin, as expected, and the largest emissions are found over western Ethiopia and western tropical Africa during March and April when it is hottest and driest (Supplementary Figs. 7–11; Supplementary note 2). Although caution should be exercised when interpreting regions smaller than 1000 km, these emission focal points are a robust feature of our analysis that extends back through the GOSAT record to 2009. We do not rule out a role for regional systematic retrieval errors^29^, but comparison to sparse independent data (Supplementary Fig. 12; Supplementary note 2) and the results from extensive sensitivity experiments (Supplementary Figs. 13–16; Supplementary note 3) support our results. The magnitude and approximate timing of the inferred seasonal cycle of net fluxes is consistent with the ORCHIDEE land surface model (Fig. 2b; Supplementary Discussion), although the model has larger uptake later in the year.

## Discussion

Compared to tropical South America there is a lower baseline for precipitation, water storage, leaf phenology, and SIF over tropical North Africa (Fig. 4b), but there is a large seasonal cycle in temperature. We find a comparatively muted seasonal cycle of HCHO columns, but a much larger seasonal cycle of DM burned (Fig. 4b), which is due to predominant grassland fuel not producing sufficient energy to be directly lofted above the boundary layer where it can be observed as HCHO. For completeness, Supplementary Figs. 17–22 show similar Hovmöller plots but for all studied land regions for the GOSAT record from 2009 to 2017. Supplementary Fig. 23 shows regionally-mean values of SIF from 2009 to 2017.

Water storage records that start in 2002 reveal successive years of drought over this region (from 2009 in Supplementary Fig. 19) that could have impacted photosynthesis^15^, land-use change^22,23^, burning extent, and possibly soil carbon stocks^24^. Fire cannot explain these emissions (Supplementary Discussion), although it has a consistent seasonal cycle (Fig. 4b). Seasonally low soil water content will limit the source from soil microbial respiration, but even a small diffuse CO_2_ flux from soils due to sustained land degradation^25^ could manifest as a seasonal net carbon source (Supplementary Discussion).

We anticipate that our findings will help re-prioritise decadal science challenges for the carbon cycle community, particularly in the context of the Paris Agreement that implicitly relies on the continued operation of natural carbon sinks. Ultimately, deeper insights into the tropical carbon cycle will only be achieved by improved integration of in situ and remote-sensed data, for the short timescales, and pan-tropical sample plot data for the longer timescales.

## Methods

### In situ CO_2_ mole fraction observations

We use discrete (weekly) air samples from 105 sites and continuous (hourly) observations from 52 sites that are part of the global atmospheric surface CO_2_ observations network. These were taken from the Observation Package (ObsPack) obspack_co2_1_GLOBALVIEWplus_v2.1_2016_09_02 data product^7^ for 2015, and from obspack_co2_1_NRT_v3.3_2017–04–19 for 2016–2017^8^; both datasets are produced by the National Oceanic and Atmospheric Administration (NOAA) Earth System Research Laboratory (ESRL).

### Satellite observations of column CO_2_

We use X_CO2_ data retrieved from the Japanese Greenhouse gases Observing SATellite (GOSAT) and the NASA Orbiting Carbon Observatory-2 (OCO-2). GOSAT^11^ was launched in January 2009 in a sun-synchronous orbit with an equatorial crossing time of 1300. We use two independent GOSAT XCO_2_ data products: v7.1 full-physics retrievals from the University of Leicester^30^ (UoL), and B7.3 of the NASA Atmospheric CO_2_ Observations from Space (ACOS^31^) activity. We use 10-s averages of the bias-corrected X_CO2_ B7.1r data product^32^ over land from OCO-2 that is the current version used by the OCO-2 science team.^33,34^

### Enhanced Vegetation Index

The Enhanced Vegetation Index (EVI) is a composite property of leaf area, chlorophyll and canopy structure^35^. We use MOD13C2 (MODIS/Terra Vegetation Indices Monthly L3 Global 0.05° CMG V006)^36^ to get EVI information. The data are only retained with pixel reliability values masked as good data (0) or marginal data (1).

### Gravity recovery and climate experiment

The Gravity Recovery and Climate Experiment (GRACE) provides information about changes in the water column^37–39^. Rooting depths of tropical terrestrial ecosystems will likely be sufficiently deep that we cannot establish a direct and immediate relationship between vegetation and changes in precipitation. Changes in gravity, due to changes in water column depth, provide a much stronger relationship with vegetation access to water. We use the surface mass change data based on the RL05 spherical harmonics from CSR (Center for Space Research at University of Texas, Austin), JPL (Jet Propulsion Laboratory) and GFZ (GeoforschungsZentrum Potsdam). The three different processing groups chose different parameters and solution strategies when deriving month-to-month gravity field variations from GRACE observations. We use the ensemble mean of the three data fields and multiply the data by the provided scaling grid. Data are available from http://grace.jpl.nasa.gov.

### Formaldehyde columns

Formaldehyde (HCHO) columns are from the Ozone Monitoring Instrument^40^ (OMI) aboard the NASA Aura satellite, which was launched in a sun-synchronous orbit in 2009. We use the NASA OMHCHOv003 data product^16^ from the NASA Data and Information Services Center, which fits HCHO slant columns in the 328.5–356.5 nm window and accounts for competing absorbers, the Ring effect, and undersampling. HCHO is a high-yield product of hydrocarbon oxidation^41,42^. It is also emitted as a direct emission from incomplete combustion^43,44^. We use the active fire data product^45^ from the NASA Moderate Resolution Imaging Spectrometer (MODIS), derived from surface thermal IR anomalies, to isolate the pyrogenic HCHO signal.

### Satellite observations of solar induced fluorescence

Satellite observations of solar induced fluorescence (SIF) are retrieved by the UoL from the GOSAT instrument^46^. SIF is a by-product of plant pigments absorbing incoming sunlight as part of photosynthesis. Of the solar radiation absorbed, ~20% is eventually dissipated as heat and typically <1–2% is emitted by SIF in the range 650–800 nm, peaking at 685–690 nm and 730–740 nm. GOSAT fits estimates of SIF at 755 nm^47^. We use the GOSAT SIF data product as a crude measure of photosynthetic capacity of regional ecosystems. We use a physically based retrieval scheme^47^ with a focus on the bias correction procedure. We use a two-stage method. First, we isolate GOSAT measurements over non-vegetated areas using the ESA CCI Land Cover product V2.0.7^48^ at 300 m resolution. Second, we apply a bias correction as an explicit function of time to ensure that instrumental effects are accounted for the entire date range of the SIF product.

### DM burned estimates

DM burned estimates are taken from the Global Fire Emission Database^49^ (GFED4). These estimates were derived by combined by satellite remote sensing observation of burned area and active fire data from MODIS.

### Atmospheric transport models and inverse methods

To describe the relationship between surface fluxes of CO_2_ and atmospheric CO_2_ we use three atmospheric transport models: (1) GEOS-Chem global 3-D chemistry transport model^50,51^ v9.02; (2) GSFC parameterised chemistry and transport model^52^ (PCTM), and (3) Laboratoire de Météorologie Dynamique (LMDZ), version LMDZ3^53^.

We run GEOS-Chem with a horizontal resolution of 4° (latitude) × 5° (longitude), driven by the GEOS-5 meteorological analyses (GEOS-FP from 2013) from the Global Modeling and Assimilation Office (GMAO) Global Circulation Model based at NASA Goddard Space Flight Center. We run the model using 47 vertical terrain-following sigma-levels that describe the atmosphere from the surface to 0.01 hPa, of which about 30 are typically below the dynamic tropopause. We use well-established emission inventories as our a priori flux estimates: (1) weekly biomass burning emissions^49;^ (2) monthly fossil fuel emissions^54,55^; (3) monthly climatological ocean fluxes^56^; and (4) three-hourly terrestrial biosphere fluxes^57^.

The GEOS-Chem model uses an ensemble Kalman Filter (EnKF) framework^18,58^ to infer CO_2_ fluxes from the ground-based or space-based measurements of atmospheric CO_2_. We use a total of 792 basis functions per month, split between 317 oceanic regions and 475 land regions. These regions are subdivisions of the 22 regions used in TransCom-3^9^. We assume a 50% uncertainty for monthly land terrestrial fluxes, and 40% for monthly ocean fluxes^49^. We assume land (ocean) a priori fluxes are correlated with a correlation length of 500 (800) km. We assume no observation error correlations, but include an additional 1.5 ppm uncertainty to the reported observation errors to account for model transport errors. We determine the terrestrial biosphere flux by subtracting the fossil fuel and cement production emission estimate (FF). This is a common approach^10,18,59^, based on the assumption knowledge of FF flux is much better than that of the natural fluxes from the land and ocean.

The LMDZ model is run using a regular horizontal resolution of 3.75° (longitude) and 1.875° (latitude), with 39 hybrid layers in the vertical. Winds are nudged towards the 6-hourly ECMWF reanalysis^60^ with a relaxation time of three hours. Fossil fuel burning emissions from the ODIAC model^54,55^, including diurnal and day-of-week variability^61^. We also use monthly ocean fluxes^56^, three-hourly biomass burning emissions (GFED 4.1 s until 2015 and GFAS afterwards), and climatological three-hourly biosphere-atmosphere fluxes taken as the 1989–2010 of a simulation of the ORganizing Carbon and Hydrology In Dynamic EcosystEms model (ORCHIDEE^62^), version 1.9.5.2.

The LMDZ CAMS inversion tool currently generates the global CO_2_ atmospheric inversion product of the Copernicus Atmosphere Monitoring Service^63,64^. The minimum of the Bayesian cost function of the inversion problem is found by an iterative process using the Lanczos version of the conjugate gradient algorithm^65^. The inferred fluxes are estimated at each horizontal grid point of the transport model with a temporal resolution of eight days, separately for day-time and night-time. The state vector of the inversion system is therefore made of a succession of global maps with 9200 grid points. Per month it gathers 73,700 variables (four day-time maps and four night-time maps). It also includes a map of the total CO_2_ columns at the initial time step of the inversion window in order to account for the uncertainty in the initial state of CO_2_. Over land, the errors of the prior biosphere-atmosphere fluxes are assumed to dominate the error budget and the covariances are constrained by an analysis of mismatches with in situ flux measurements: temporal correlations on daily mean net carbon exchange (NEE) errors decay exponentially with a length of one month but night-time errors are assumed to be uncorrelated with daytime errors; spatial correlations decay exponentially with a length of 500 km; standard deviations are set to 0.8 times the climatological daily-varying heterotrophic respiration flux simulated by ORCHIDEE with a ceiling of 4 gC/m^2^/day. Over a full year, the total 1-sigma uncertainty for the prior land fluxes amounts to about 3.0 GtC/yr. The error statistics for the open ocean correspond to a global air-sea flux uncertainty about 0.5 GtC/yr and are defined as follows: temporal correlations decay exponentially with a length of one month; unlike land, daytime and night-time flux errors are fully correlated; spatial correlations follow an e-folding length of 1000 km; standard deviations are set to 0.1 gC/m^2^/day. Land and ocean flux errors are not correlated.

PCTM is run at a horizontal resolution of 2.0° (latitude) × 2.5° (longitude) with 40 hybrid sigma levels in the vertical, driven by winds, surface pressure, and vertical mixing parameters from NASA MERRA2 reanalyses^66^. A priori fluxes for gross primary productivity, gross respiration, wildfires and biofuel emissions are taken from CASA-GFED3 land biosphere model^49,67,68^. Fossil fuel burning emissions from the ODIAC model^54,55^, including diurnal and day-of-week variability^61^, and air-sea CO_2_ fluxes from three different sources: the NASA Ocean and Biosphere Model (NOBM^69^), and two CO_2_ climatological flux products^56,70^.

The CSU inversion scheme uses a variational data assimilation approach^71,72^. A priori CO_2_ fluxes are run forward through PCTM at a 2.0° × 2.5° (lat/lon) resolution, with the resulting model-measurement residuals used in a 6.7° × 6.7° version of PCTM to estimate weekly flux corrections (no day/night split); no correlations in space or time are assumed. This configuration results in 54 × 27 × 4.33 ≈ 6300 monthly flux corrections being solved. The adjoint of PCTM, forced with the measurement mismatches, generates the gradient to the Bayesian cost function; this is used in a BFGS approach (pre-conditioned with the a priori flux uncertainties) to descend to the minimum, giving the optimal fluxes.

## Supplementary information

Supplementary Final

Peer Review File

## Data Availability

GOSAT V7.1 and SIF data are available from University of Leicester. OCO-2 retrievals were produced by the OCO-2 project at the Jet Propulsion Laboratory, California Institute of Technology, and obtained from the OCO-2 data archive maintained at the NASA Goddard Earth Science Data and Information Services Center. All correlative data are also freely available from NASA data repositories. The NOAA in situ data are freely available from the ESRL website (https://www.esrl.noaa.gov/gmd/ccgg/trends/data.html).

## Code availability

The community-led GEOS-Chem model of atmospheric chemistry and model is maintained centrally by Harvard University (http://acmg.seas.harvard.edu/geos/), and is available on request. The ensemble Kalman filter code is publicly available as PyOSSE (https://www.nceo.ac.uk/data-tools/atmospheric-tools/). The CAMS inversion system is available on simple request from FC. For access to PCTM, please contact DB.
