# Supplementary material for: Net carbon emissions from African biosphere dominate pan-tropical atmospheric CO2 signal
Source: Nat Commun. 2019 Aug 13;10:3344. doi: 10.1038/s41467-019-11097-w (PMC6692308; doi:10.1038/s41467-019-11097-w)
Supplement: Supplementary file 1 — Supplementary Final [file 41467_2019_11097_MOESM1_ESM.pdf]

Supplementary Information for Net carbon emissions from African land biosphere dominate pan-tropical atmospheric CO_2 signal

Paul I. Palmer†, Liang Feng[1], David Baker[1], Frédéric Chevallier[1], and Hartmut Bösch, Peter Somkuti[1]

†Corresponding author: paul.palmer@ed.ac.uk

June 12, 2019

Supplementary Figures

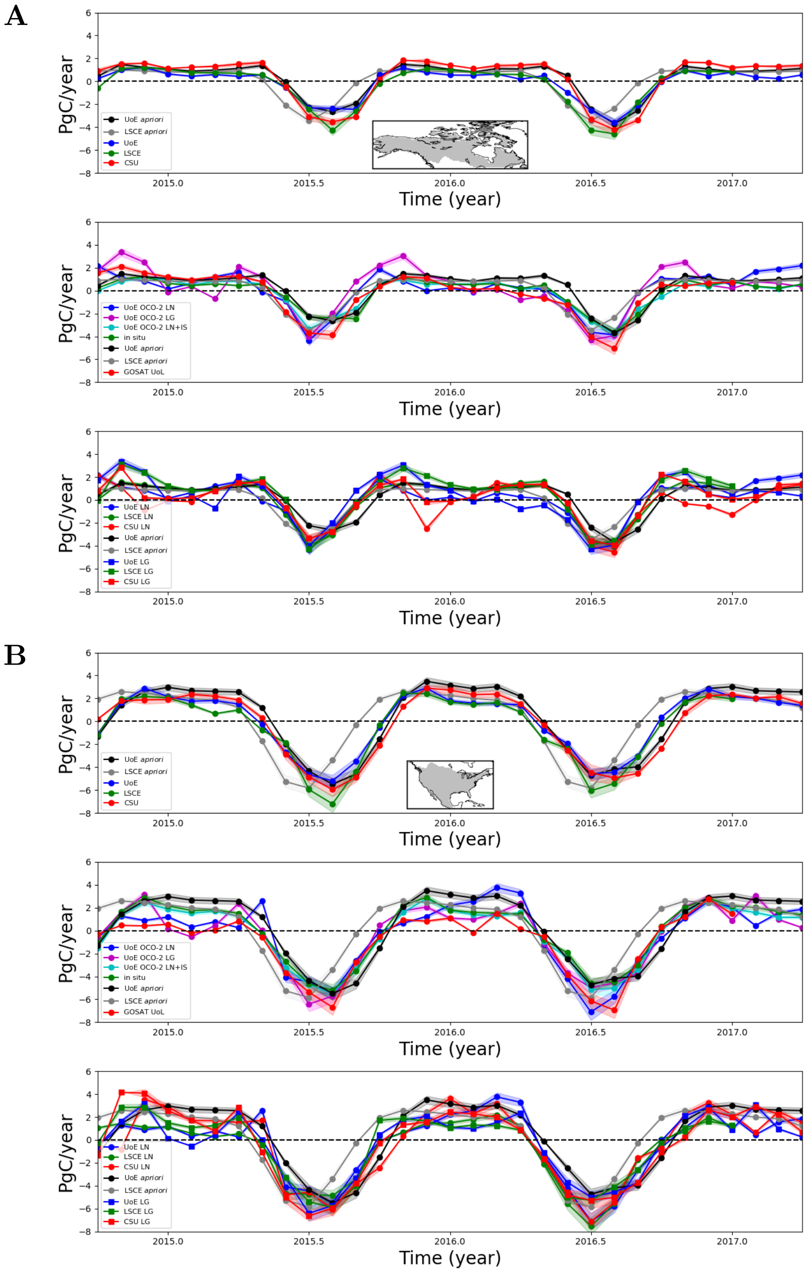

Supplementary Figure 1: Monthly *a priori* and *a posteriori* CO_2 fluxes (expressed as PgC/yr, mid-2014 to 2017) from (A) Boreal North America and (B) and Temperature North America. Each panel is comprised of three sub-panels: 1) *a priori* and *a posteriori* CO_2 net fluxes inferred from *in situ* data using three independent atmospheric transport models; 2) CO_2 net fluxes inferred from GOSAT X_{CO2} data, OCO-2 land nadir (LN) and land glint (LG) X_{CO2} data, or *in situ* data using the GEOS-Chem atmospheric transport model; and 3) CO_2 net fluxes inferred from OCO-2 LN and LG data using three independent atmospheric transport models. The geographical regions are shown inset of each top sub-panel.

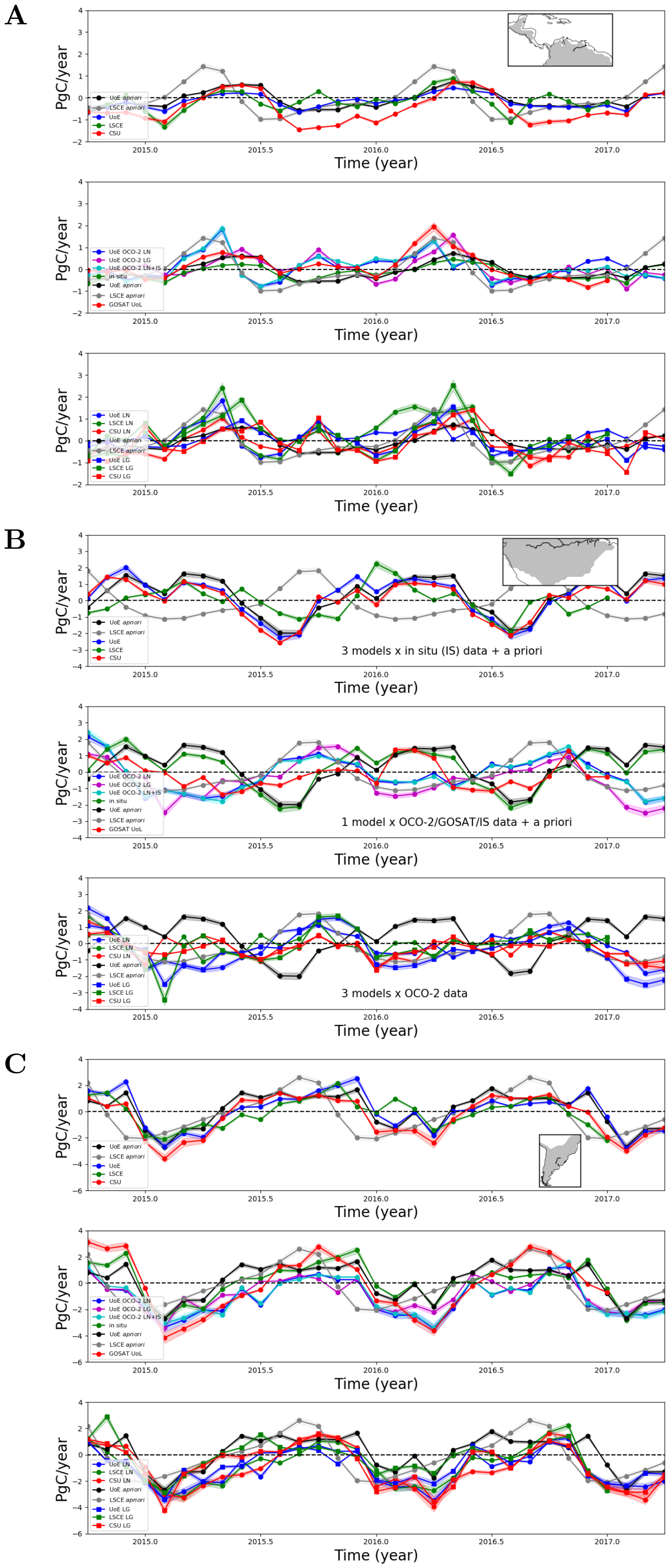

Supplementary Figure 2: As Figure 1 but for (A) northern tropical South America, (B) southern tropical South America, and (C) South America temperate.

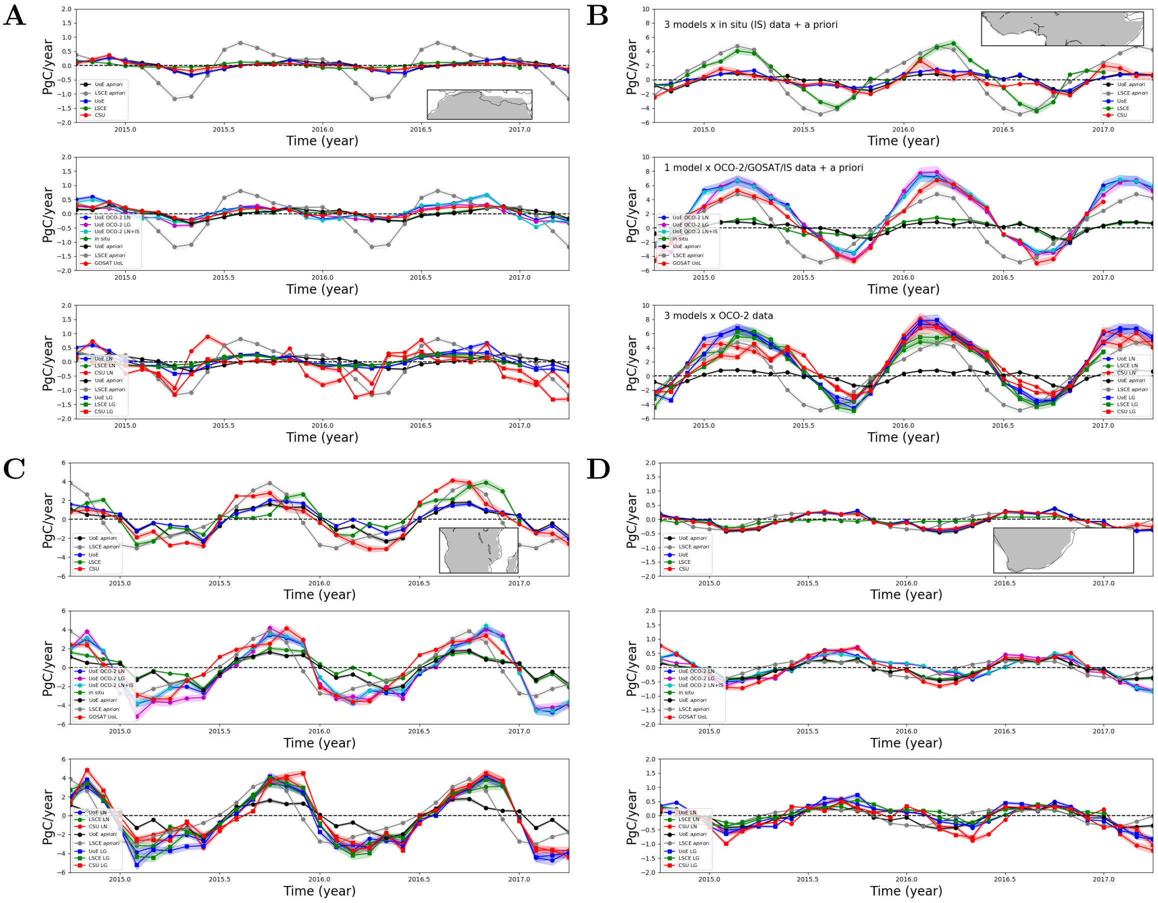

Supplementary Figure 3: As Figure 1 but for (A) northern extratropical Africa, (B) northern Tropical Africa, (C) southern Tropical Africa, and (D) southern extratropical Africa.

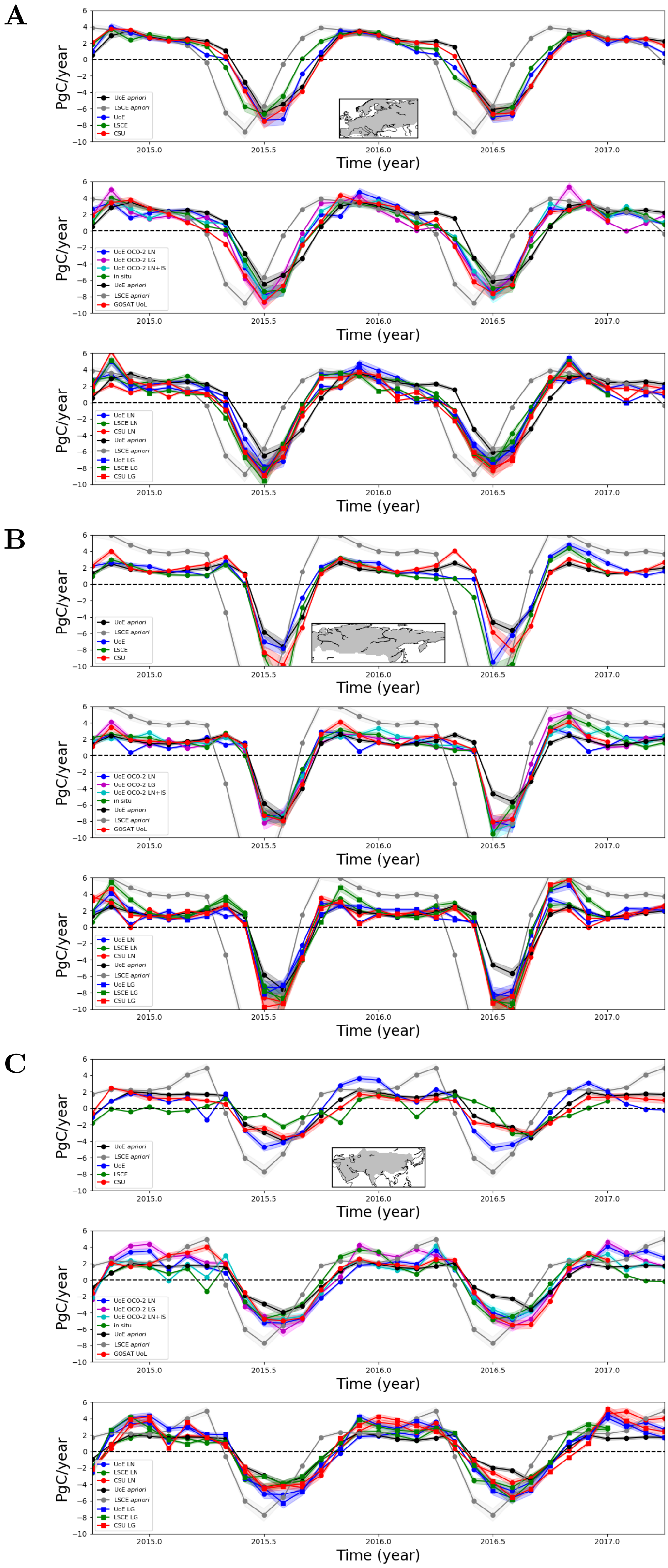

Supplementary Figure 4: As Figure 1 but for (A) Europe, (B) boreal Eurasia, and (C) temperate Eurasia.

A

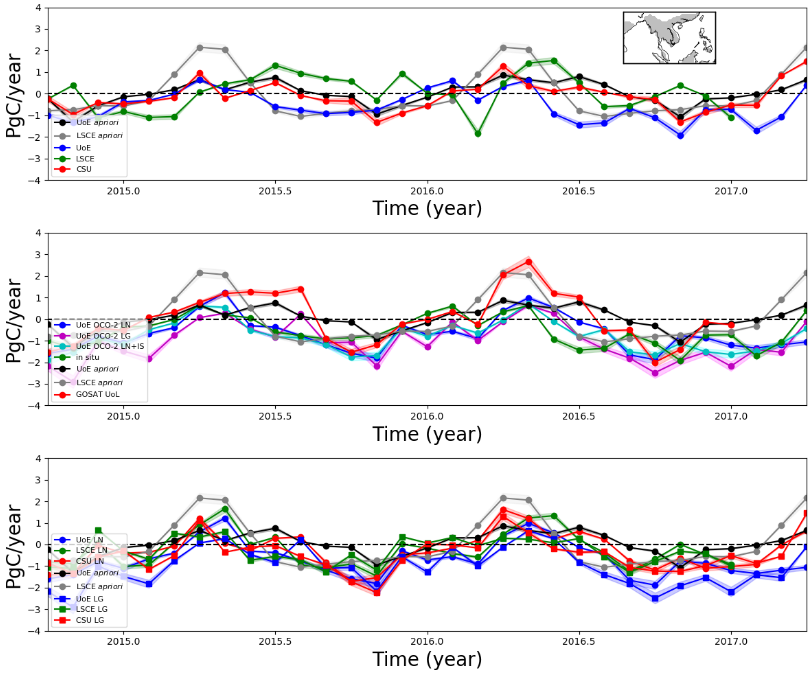

B

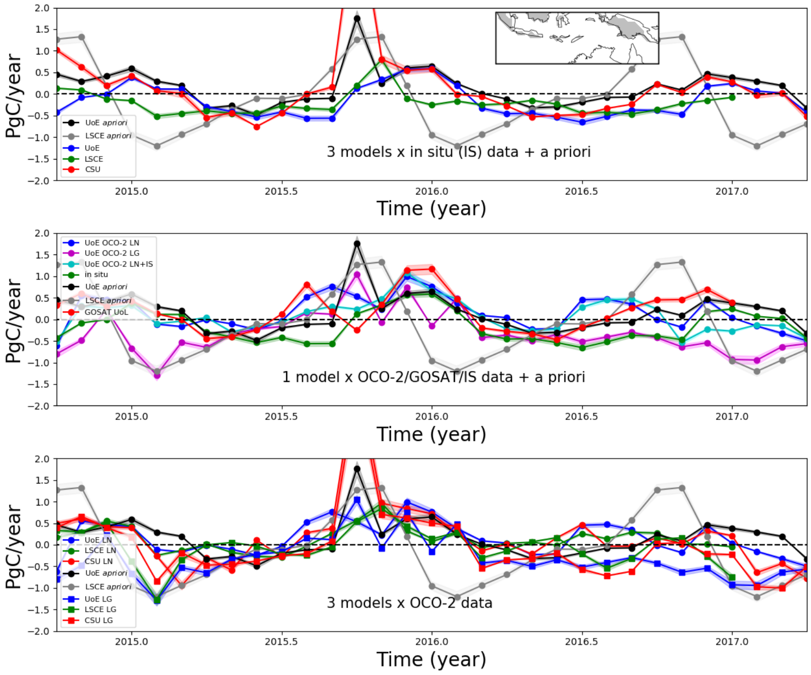

Supplementary Figure 5: As Figure 1 but for (A) northern tropical Asia and (B) southern tropical Asia.

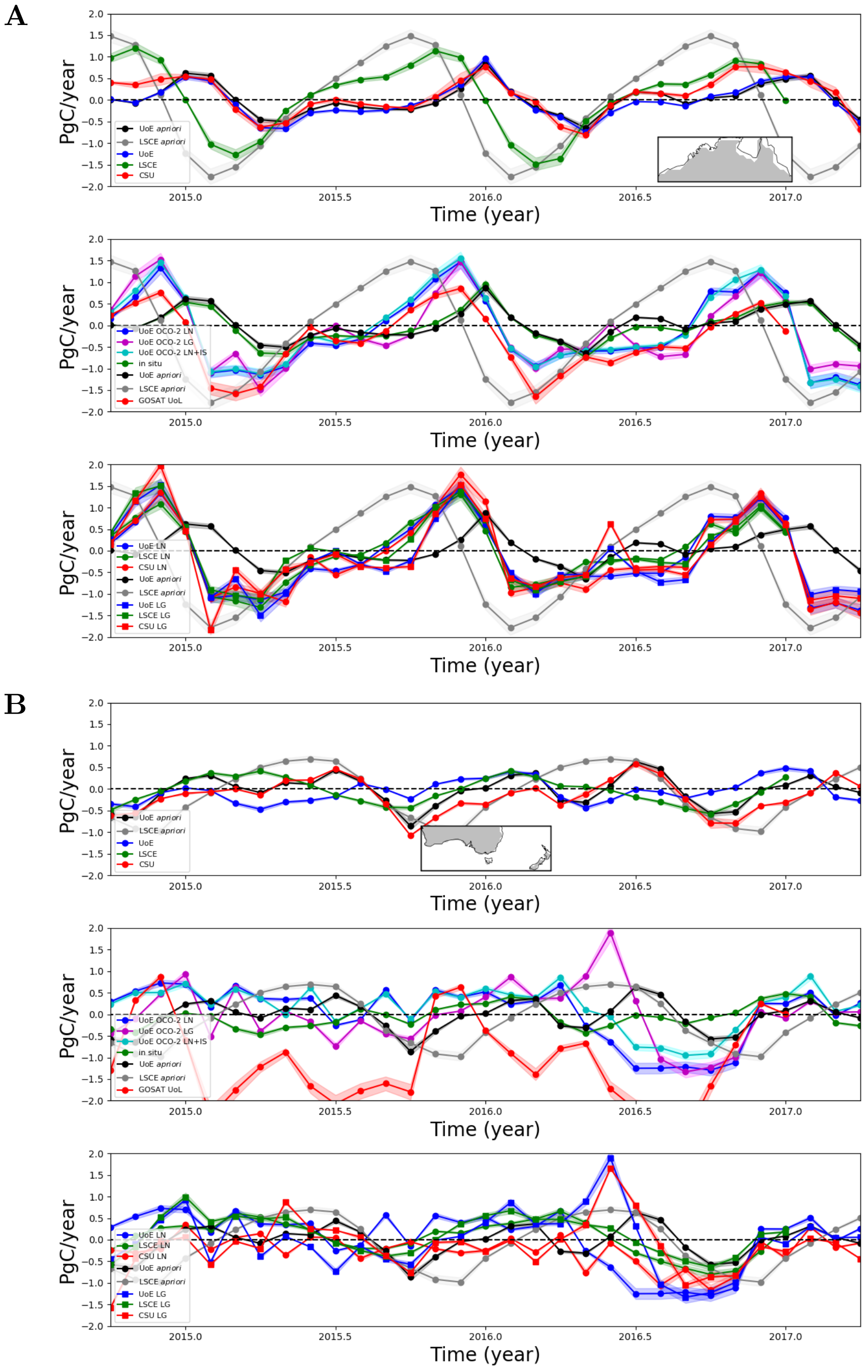

Supplementary Figure 6: As Figure 1 but for (A) tropical Australia and (B) temperate Australia.

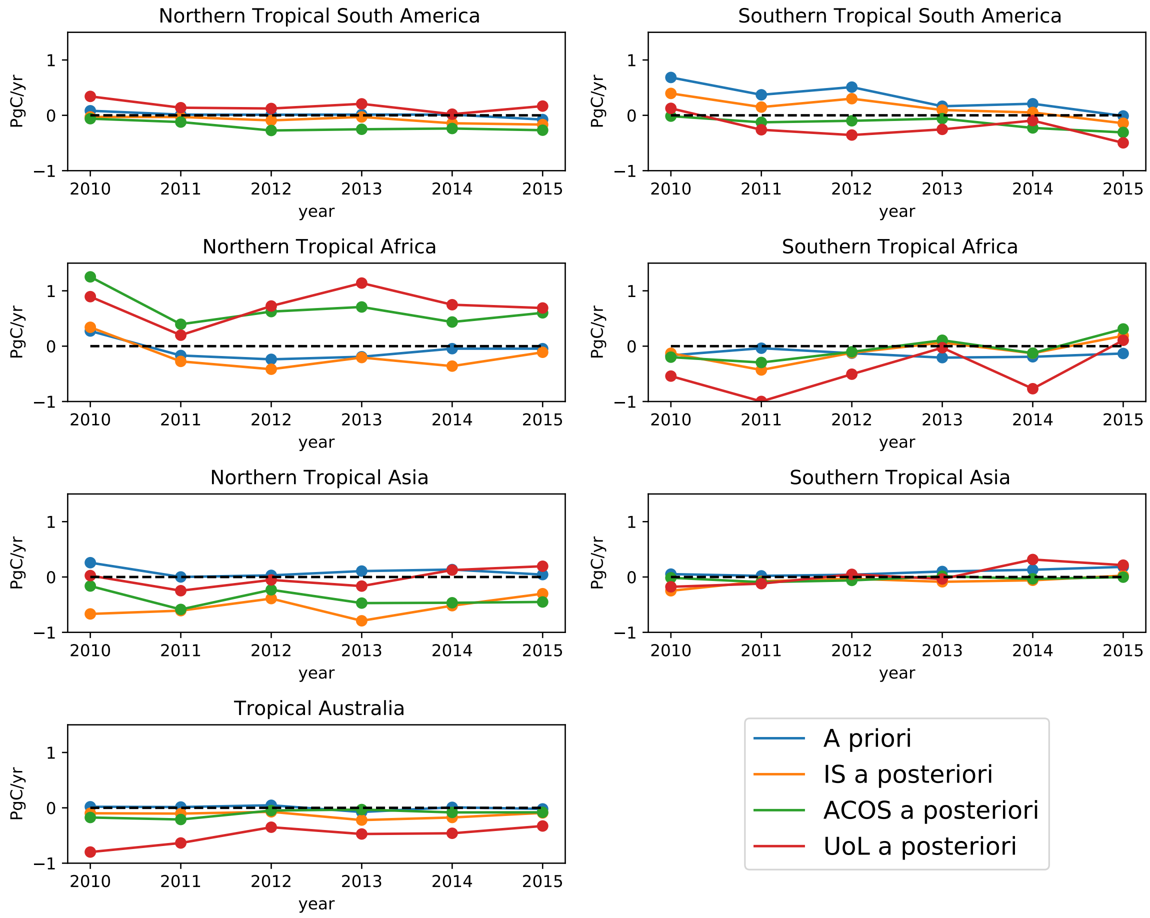

Supplementary Figure 7: Summary of annual *a priori* and *a posteriori* net carbon flux estimates (PgC/yr) from tropical geographical regions, 2010 to 2015.

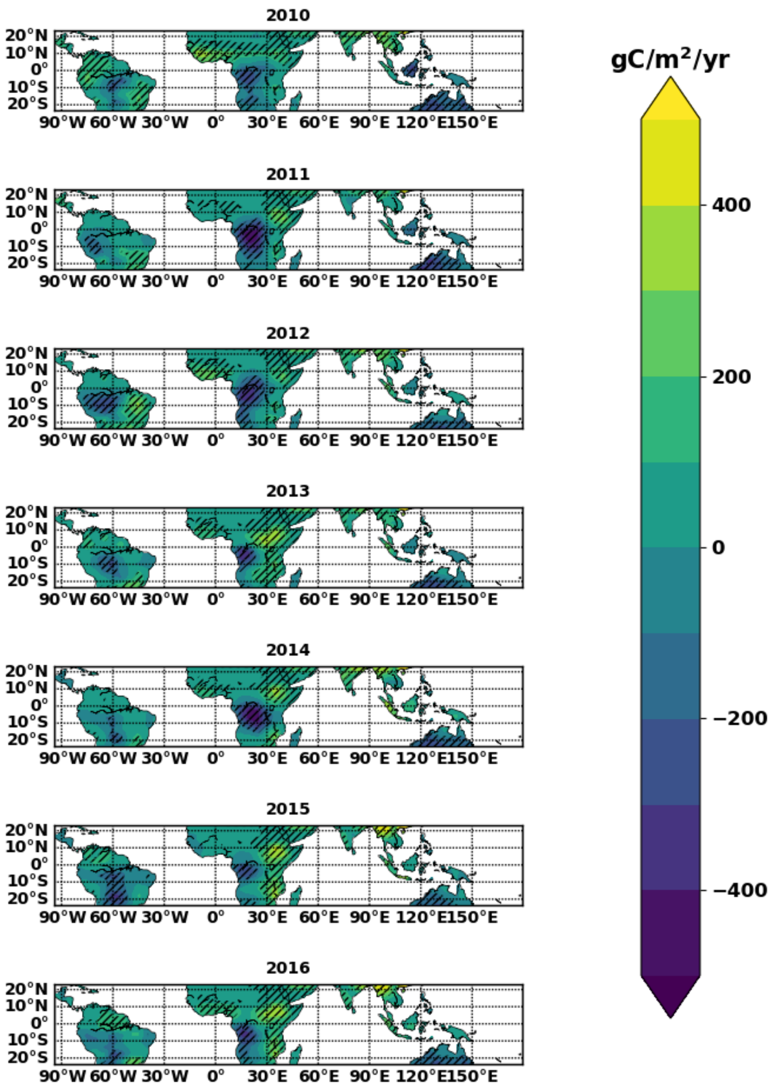

Supplementary Figure 8: Annual mean distribution of CO_2 fluxes (gC/m^2/yr) over tropical continents inferred from GOSAT X_{CO2} data, 2010–2016. Hatching denotes regions where the absolute value of the *a posteriori* flux estimate is larger than 1.25 times its corresponding uncertainty.

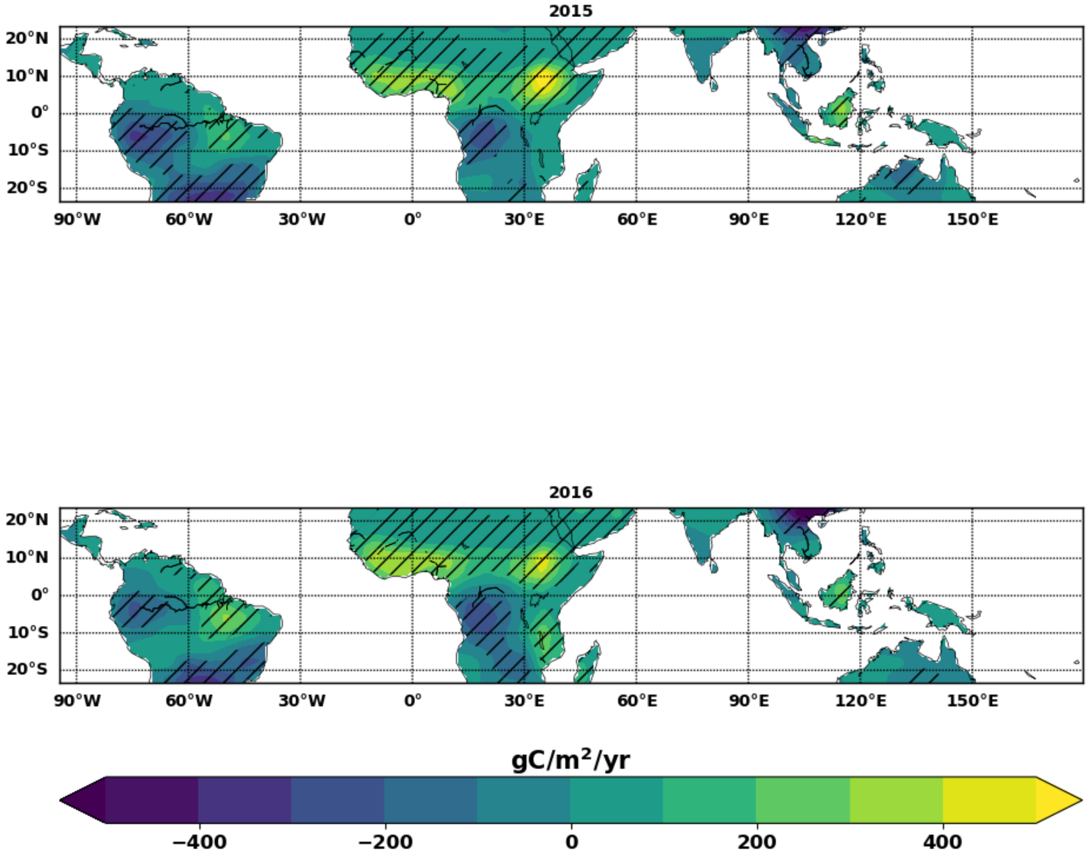

Supplementary Figure 9: Annual mean distribution of CO_2 fluxes (gC/m^2/yr) over tropical continents inferred from OCO-2 X_{CO2} data, 2015–2016. Hatching denotes regions where the absolute value of the *a posteriori* flux estimate is larger than 1.25 times its corresponding uncertainty.

DJF

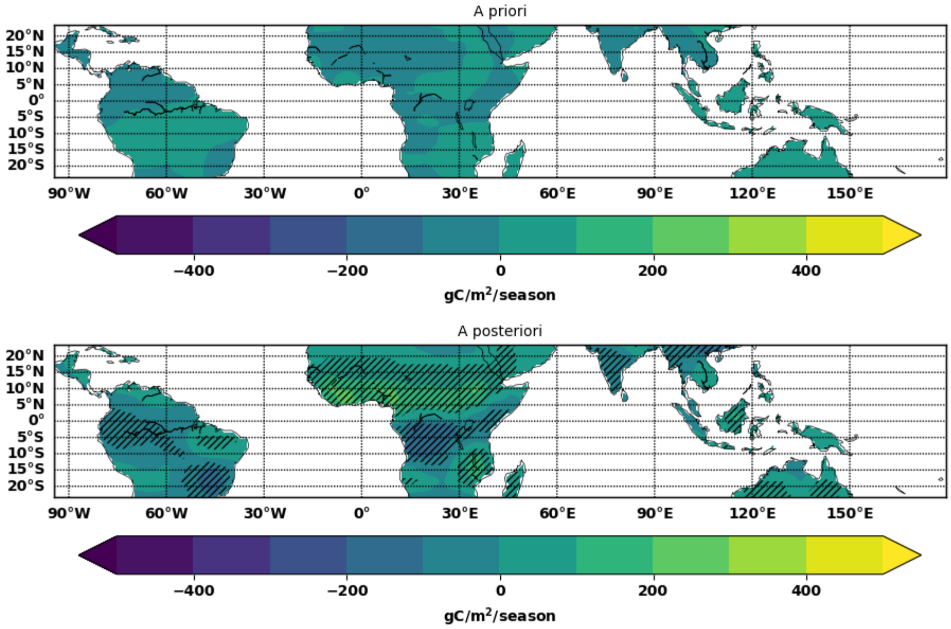

MAM

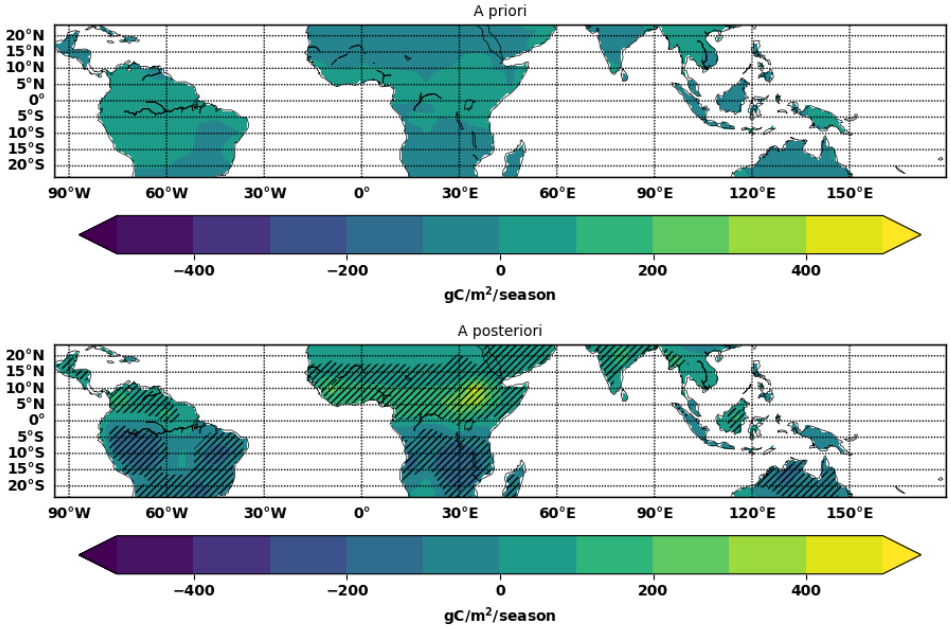

Supplementary Figure 10: Seasonal DJF and MAM distributions of CO_2 fluxes (gC/m^2/season) over tropical continents inferred from OCO-2 X_{CO2} data from 2015. Hatching denotes regions where the absolute value of the *a posteriori* flux estimate is larger than 1.25 times its corresponding uncertainty.

JJA

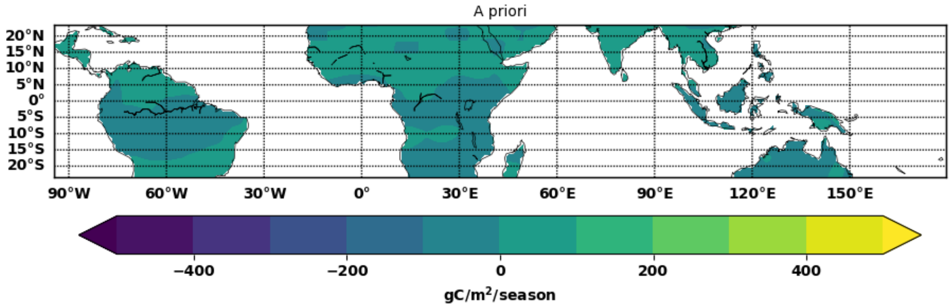

SON

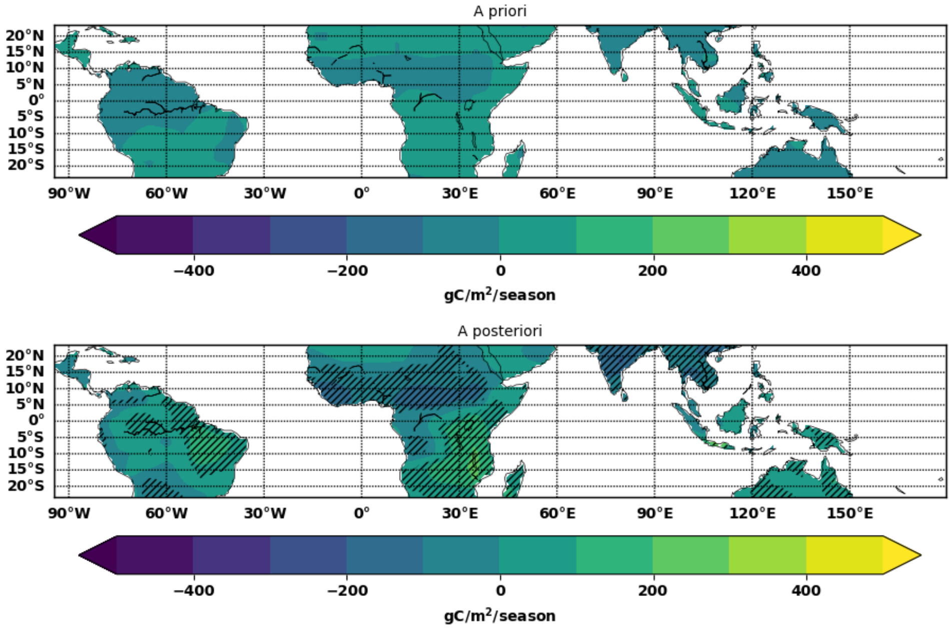

Supplementary Figure 11: As Figure 10 but for JJA and SON 2015.

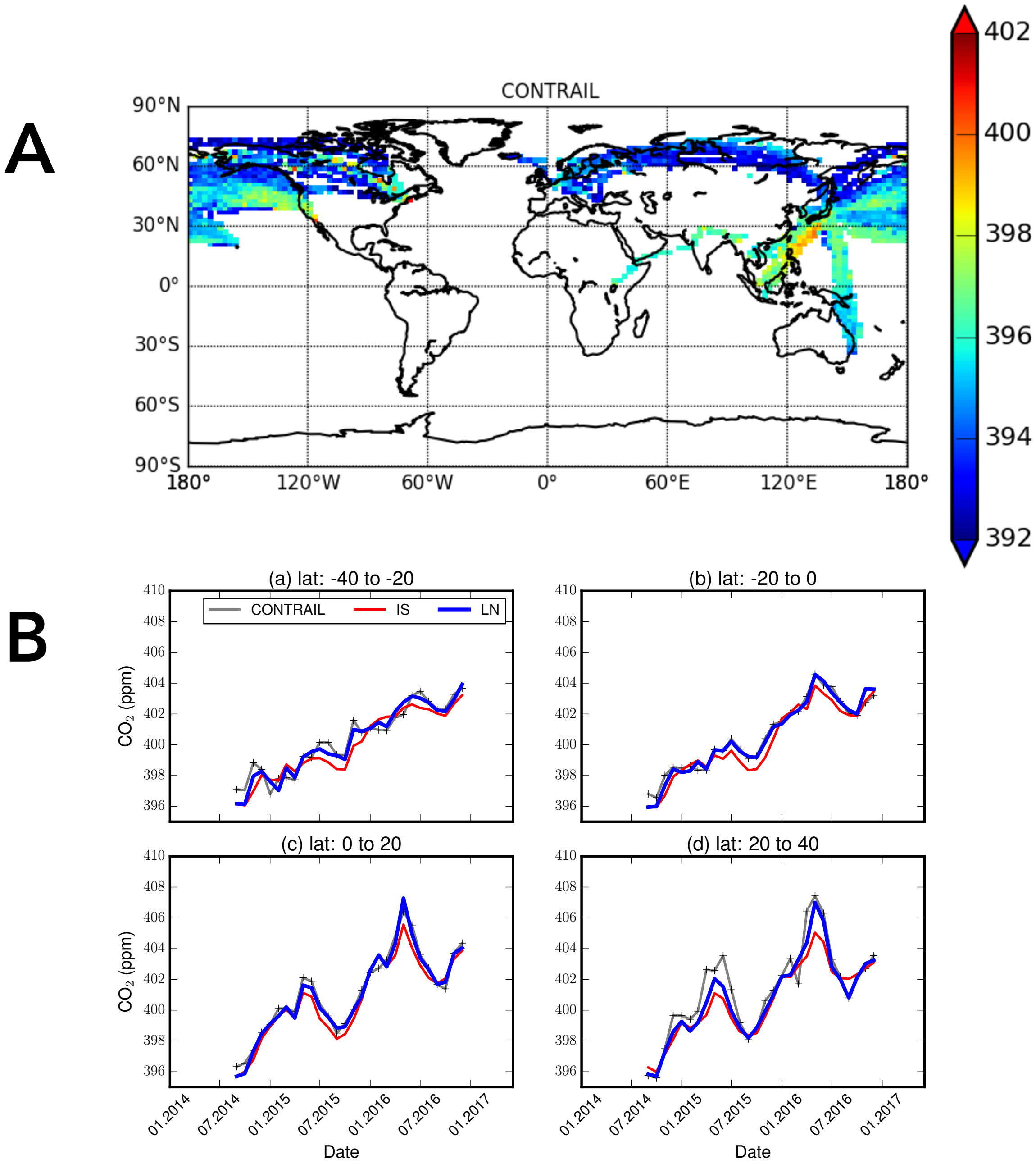

Supplementary Figure 12: (A) Annual distribution of CO_2 mole fraction measurements collected by CONTRAIL described on a $4°\times5°$ grid. Panels (B) and (C) compare CONTRAIL zonal-mean mole fraction data from 2015–2016 with *a posteriori* CO_2 fluxes inferred from OCO-2 X_{CO2} and ground-based data, respectively.

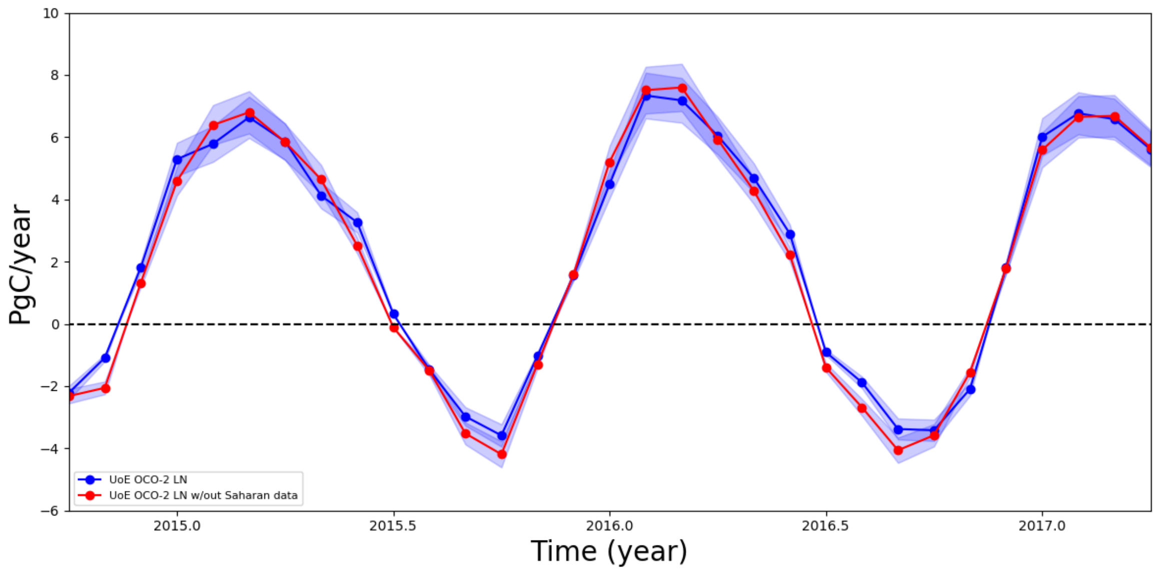

Supplementary Figure 13: Monthly *a posteriori* CO_2 fluxes over tropical north Africa, expressed as PgC/yr, inferred from land nadir (LN) data from OCO-2 with (blue) and without (red) coverage over the Sahara desert.

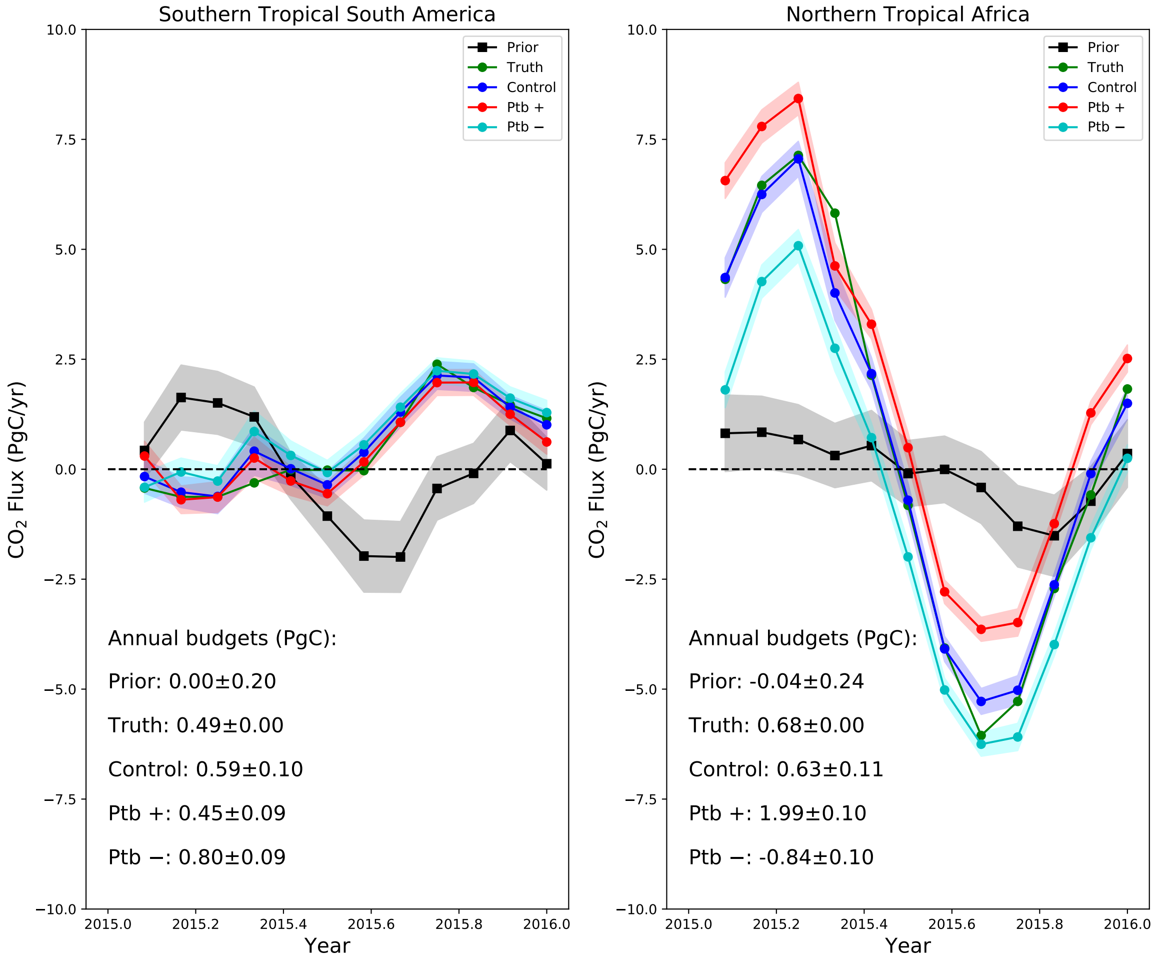

Supplementary Figure 14: Synthetic monthly CO_2 fluxes (PgC/yr) over (left) tropical South America and (right) tropical North Africa inferred from OCO-2 X_{CO2} data. The true state is denoted by the green line and the *a priori* is denoted by the black line. The *a posteriori* flux estimates are denoted by blue, red, and cyan lines that correspond to the control calculation and two perturbation calculations ($\pm$ 1 ppm systematic error), respectively. The coloured envelopes represent the 1-σ uncertainties on the *a priori* and *a posteriori*, respectively.

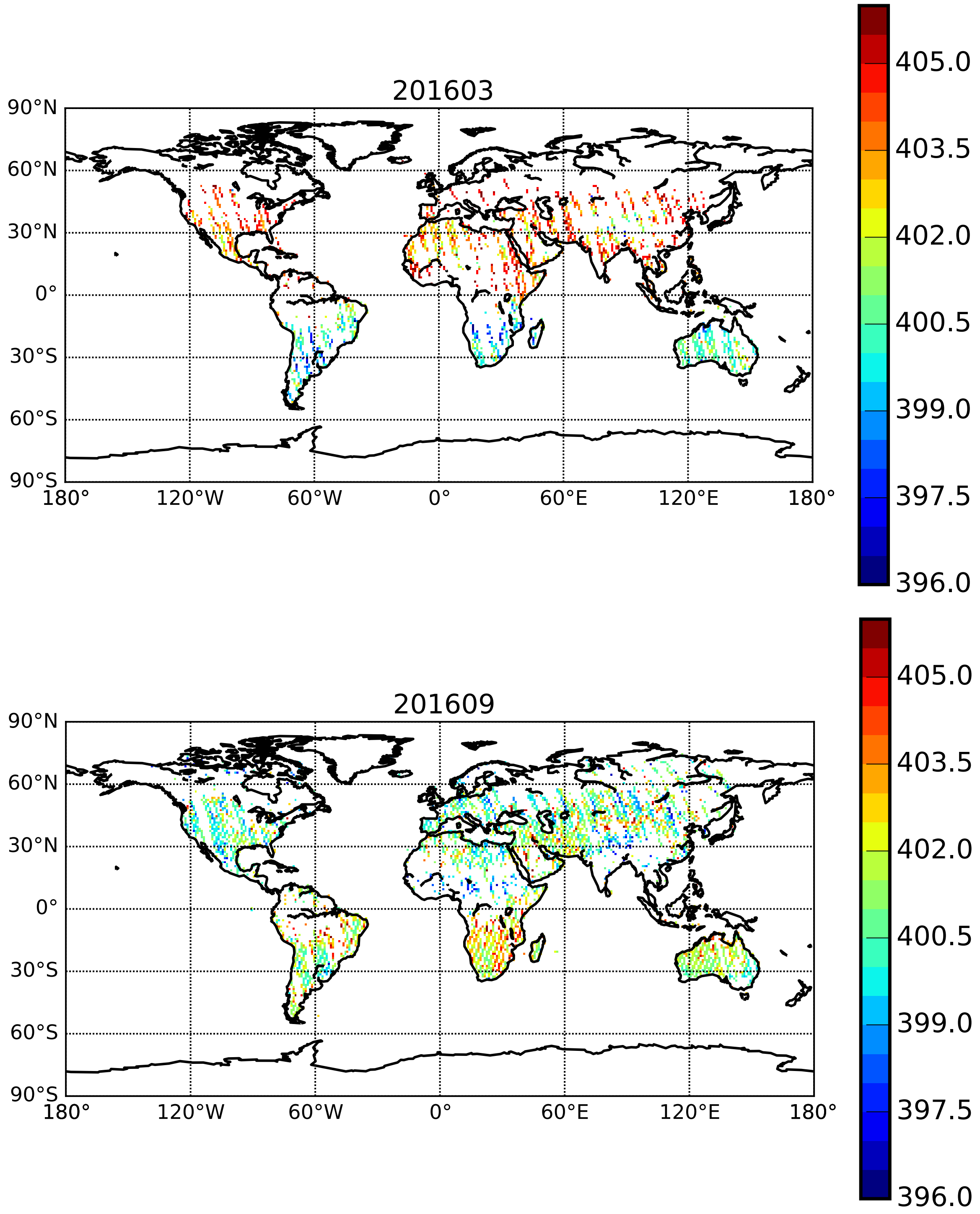

Supplementary Figure 15: Monthly spatial distribution and value of X_{CO2} data, averaged on a regular 1°×1° grid, collected by OCO-2 for (top panel) March 2016 and (bottom panel) September 2016.

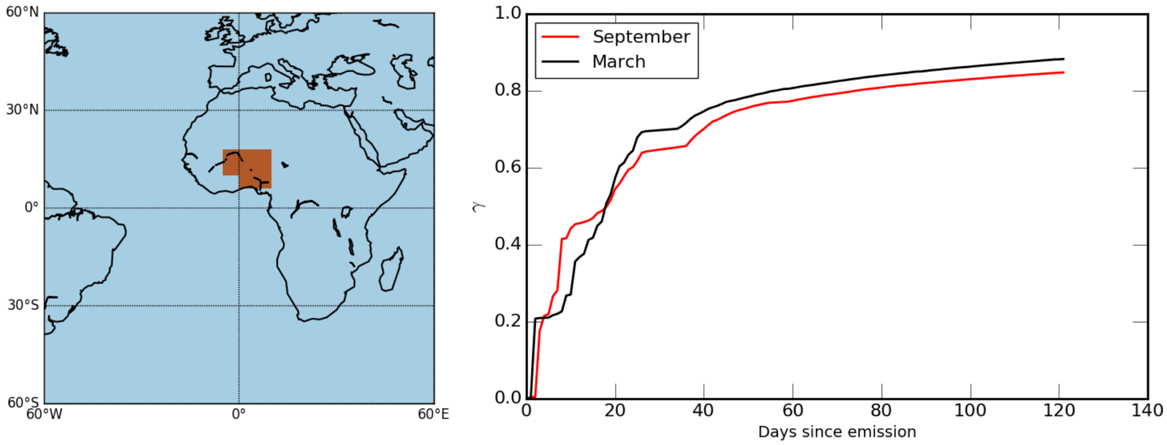

Supplementary Figure 16: (left) Geographical region for our example sampling bias calculation (denoted in red) and (right) the monthly flux error reduction associated with measurements accumulated days since the flux being estimated.

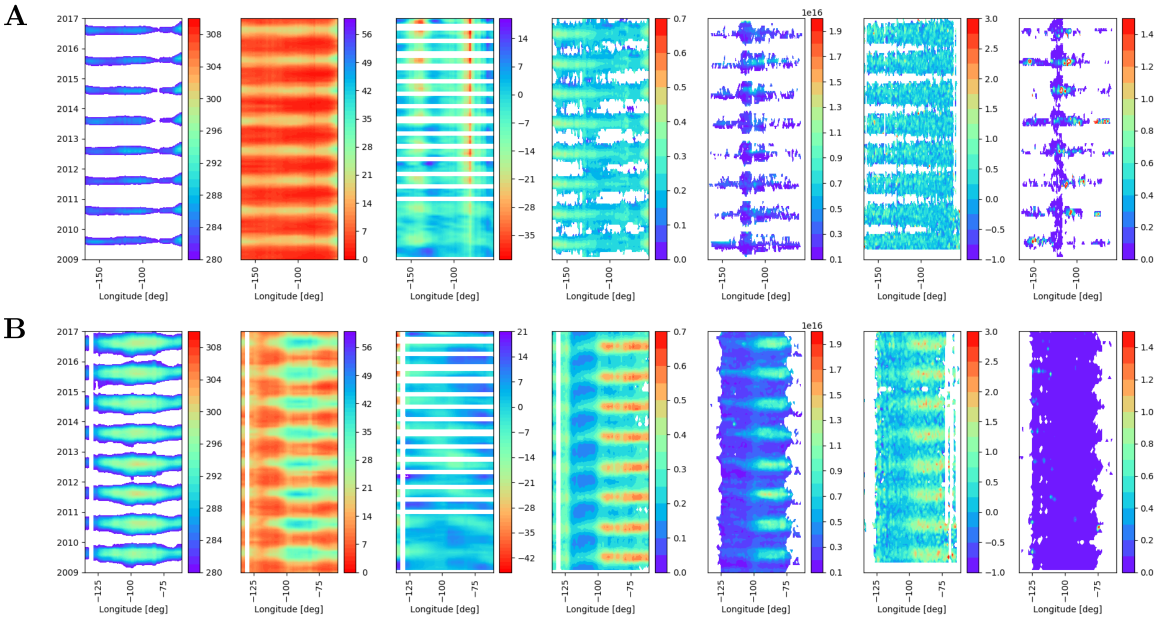

Supplementary Figure 17: Correlative data over (A) boreal North America and (B) temperate North America. The panels are from left to right: surface temperature (K), precipitation (mm/m2/day), water storage (cm), elevated vegetation index (unitless), HCHO columns (molec/cm^2) filtered for fire activity using MODIS fire counts, GOSAT solar induced fluoroscence (SIF, mW/m^2/sr/nm), and dry matter (DM) burned (kg/DM/m^2/month).

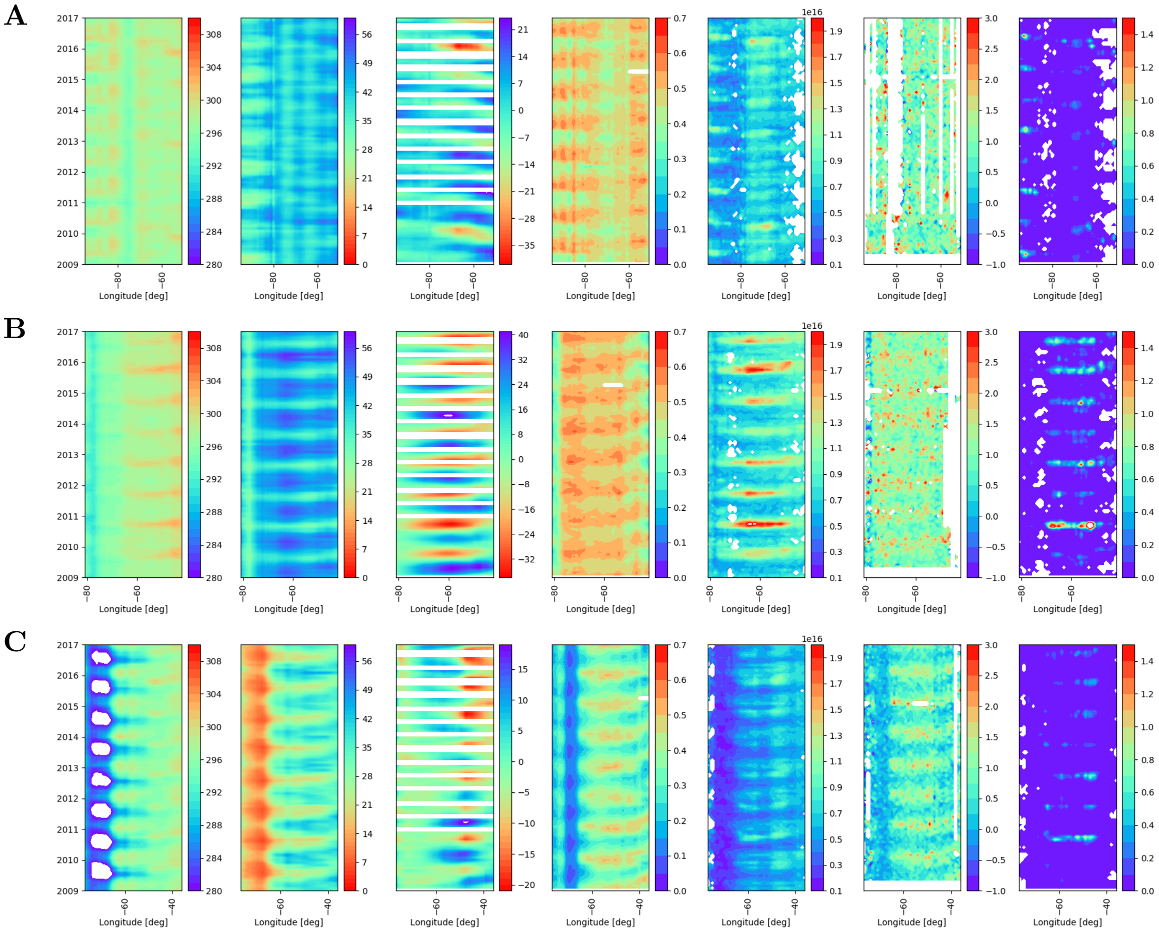

Supplementary Figure 18: As Figure 17 but for (A) northern tropical South America, (B) southern tropical South America, and (C) temperature South America.

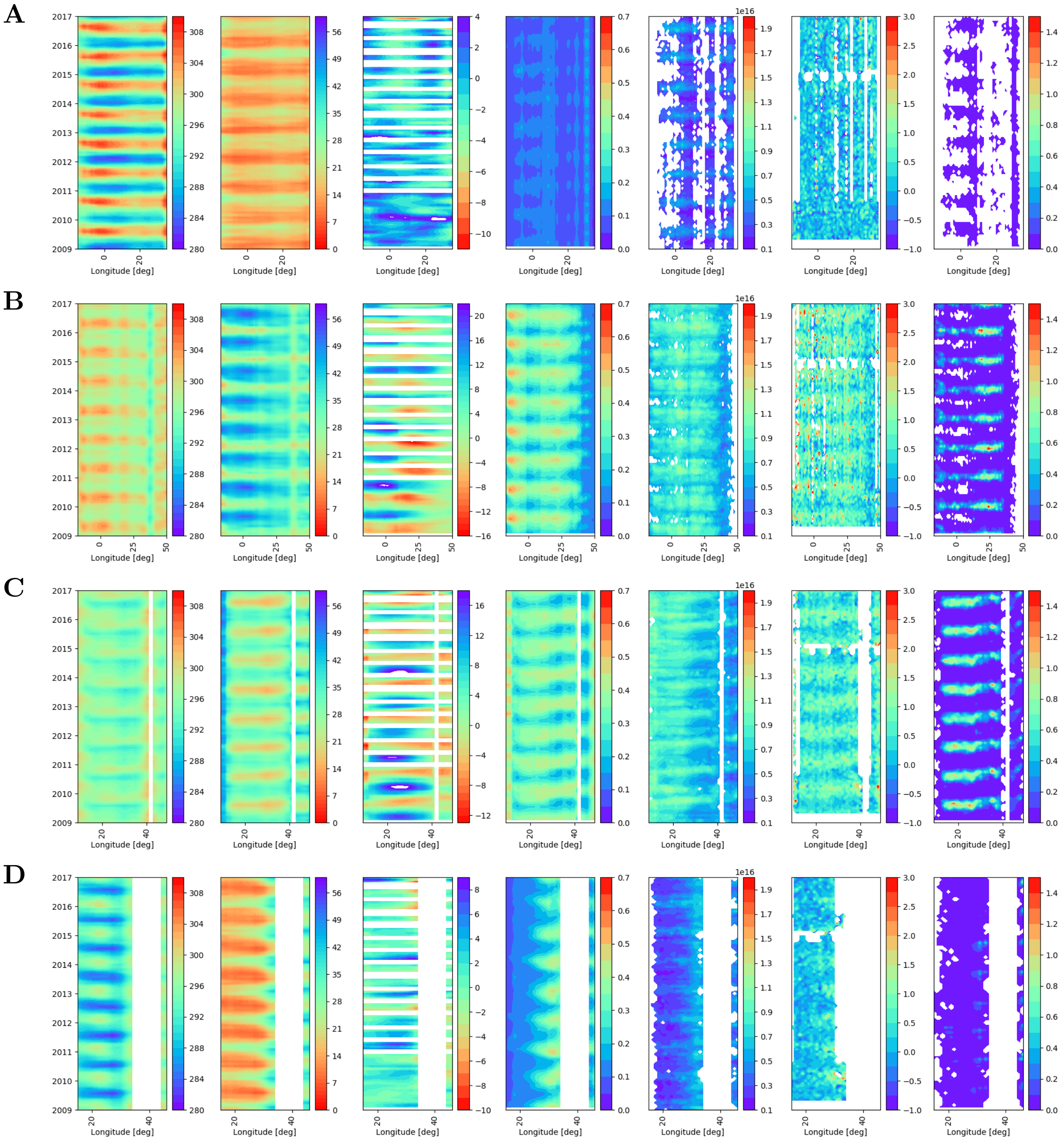

Supplementary Figure 19: As Figure 17 but for (A) northern extratropical Africa, (B) northern Tropical Africa, (C) southern Tropical Africa, and (D) southern extratropical Africa.

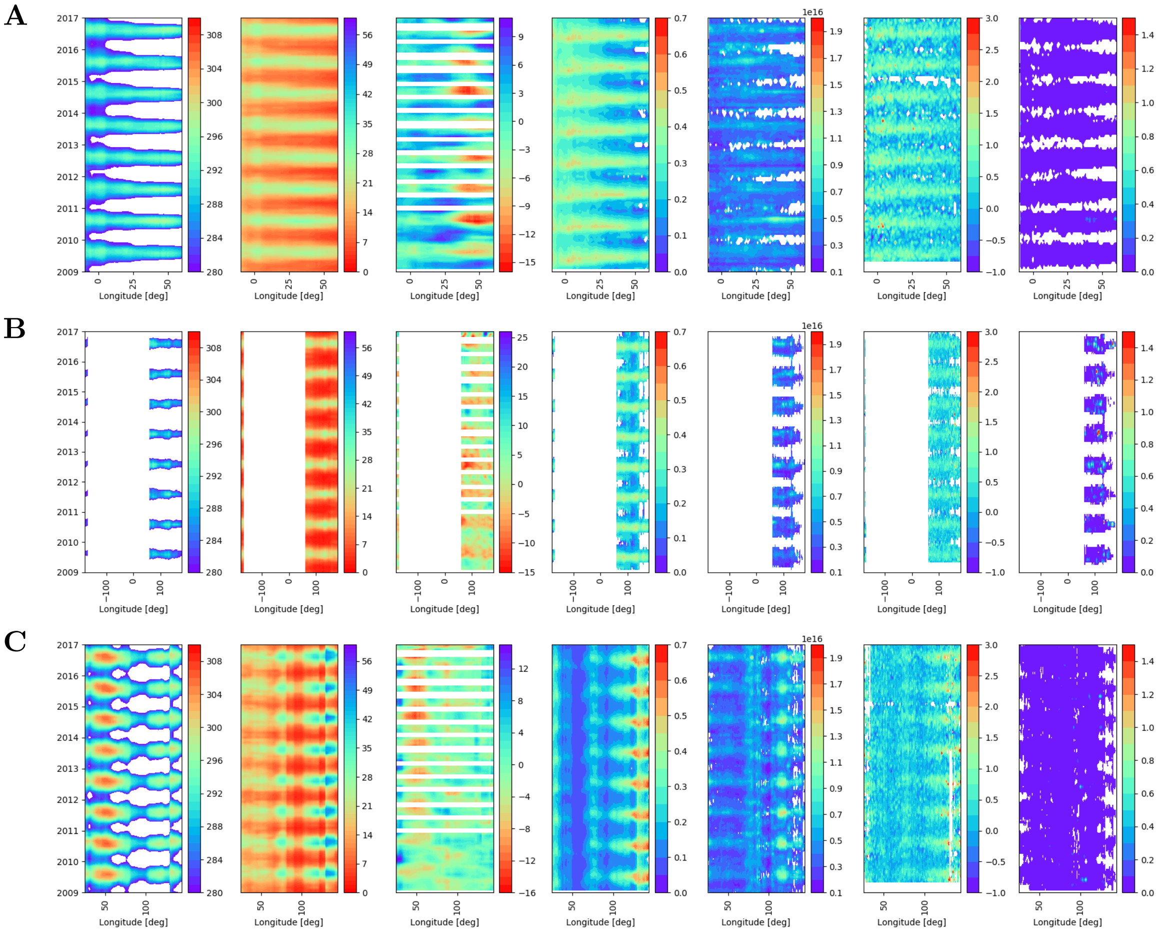

Supplementary Figure 20: As Figure 17 but for (A) Europe, (B) boreal Eurasia, and (C) temperate Eurasia.

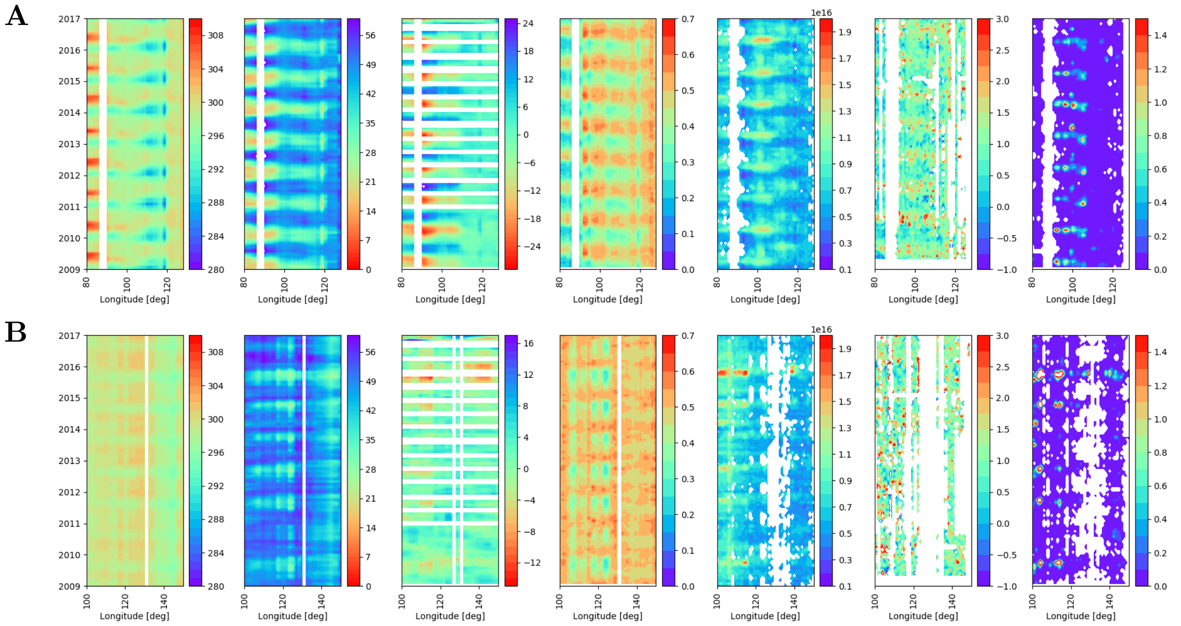

Supplementary Figure 21: As Figure 17 but for (A) northern tropical Asia and (B) southern tropical Asia.

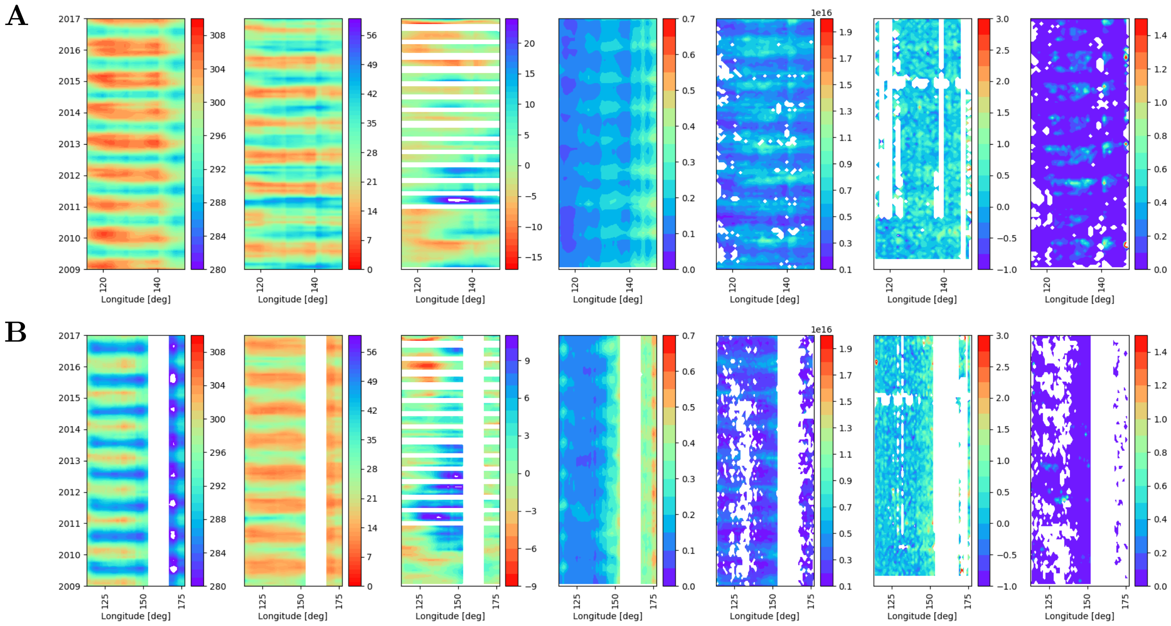

Supplementary Figure 22: As Figure 17 but for (A) tropical Australia and (B) temperate Australia.

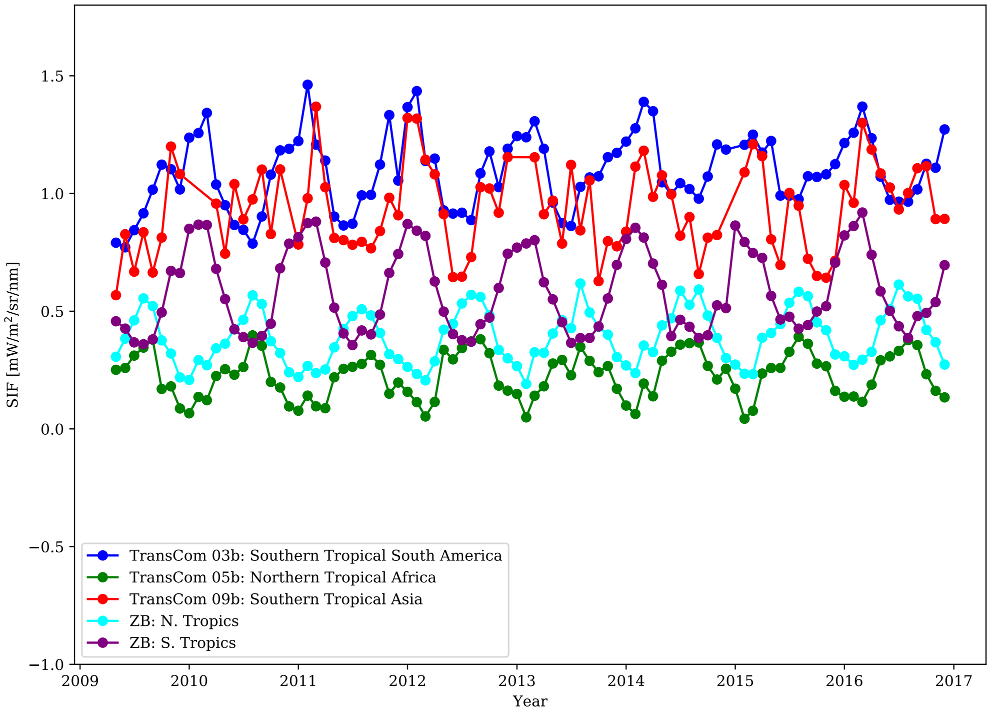

Supplementary Figure 23: Monthly SIF values (mW/m^2/sr/nm) averaged over key tropical regions.

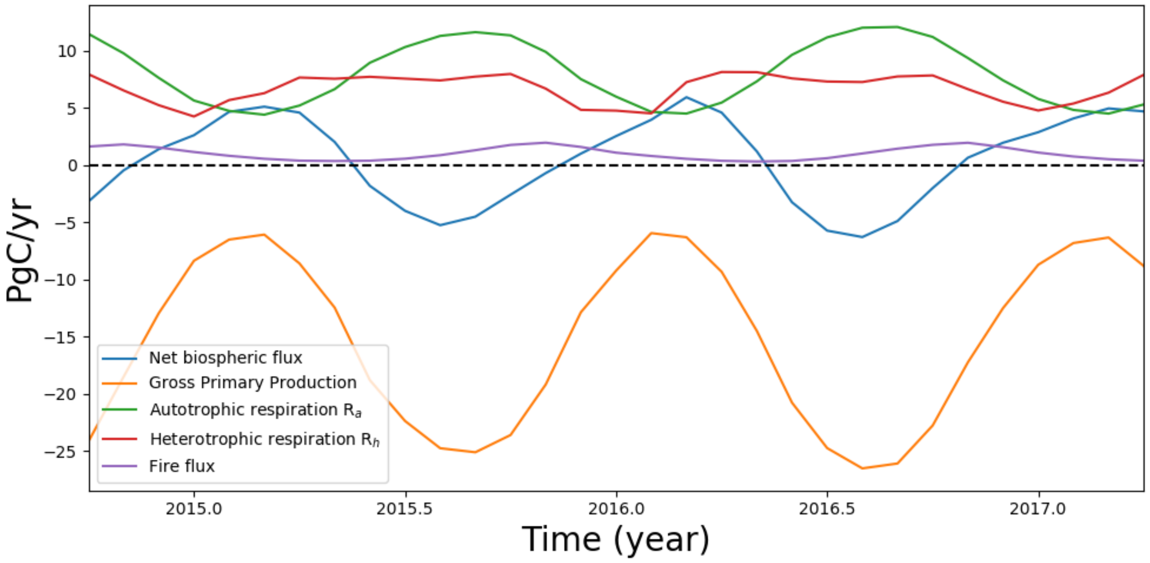

Supplementary Figure 24: Individual monthly carbon flux contributions over northern Tropical Africa (PgC/yr) taken from the ORCHIDEE model [18], 2015–2016: gross primary production (GPP), autotrophic R_a and heterotrophic R_h respiration, fire flux, and the corresponding net biospheric flux (GPP+R_a+R_h+fire). A positive value for GPP represents a loss of carbon from the atmosphere and a negative carbon flux.

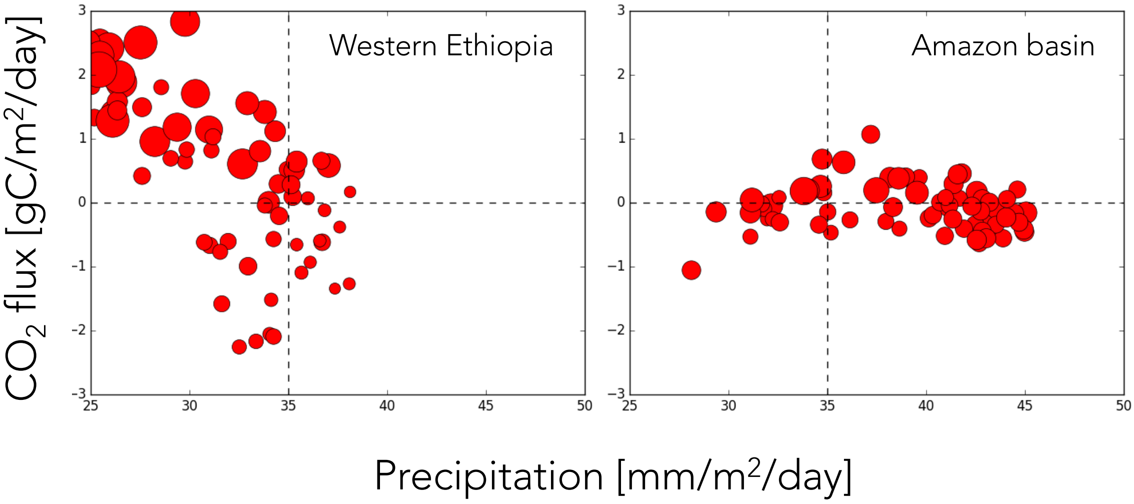

Supplementary Figure 25: Scatterplot of *a posteriori* CO_2 flux [gC/m^2/day] inferred from GOSAT and precipitation [mm/m^2/day] for (left) western Ethiopia (4–14°N, 33–38°E) and (right) the Amazon basin (0–20°S, 50–80°W). Larger red filled circles denote higher temperatures.

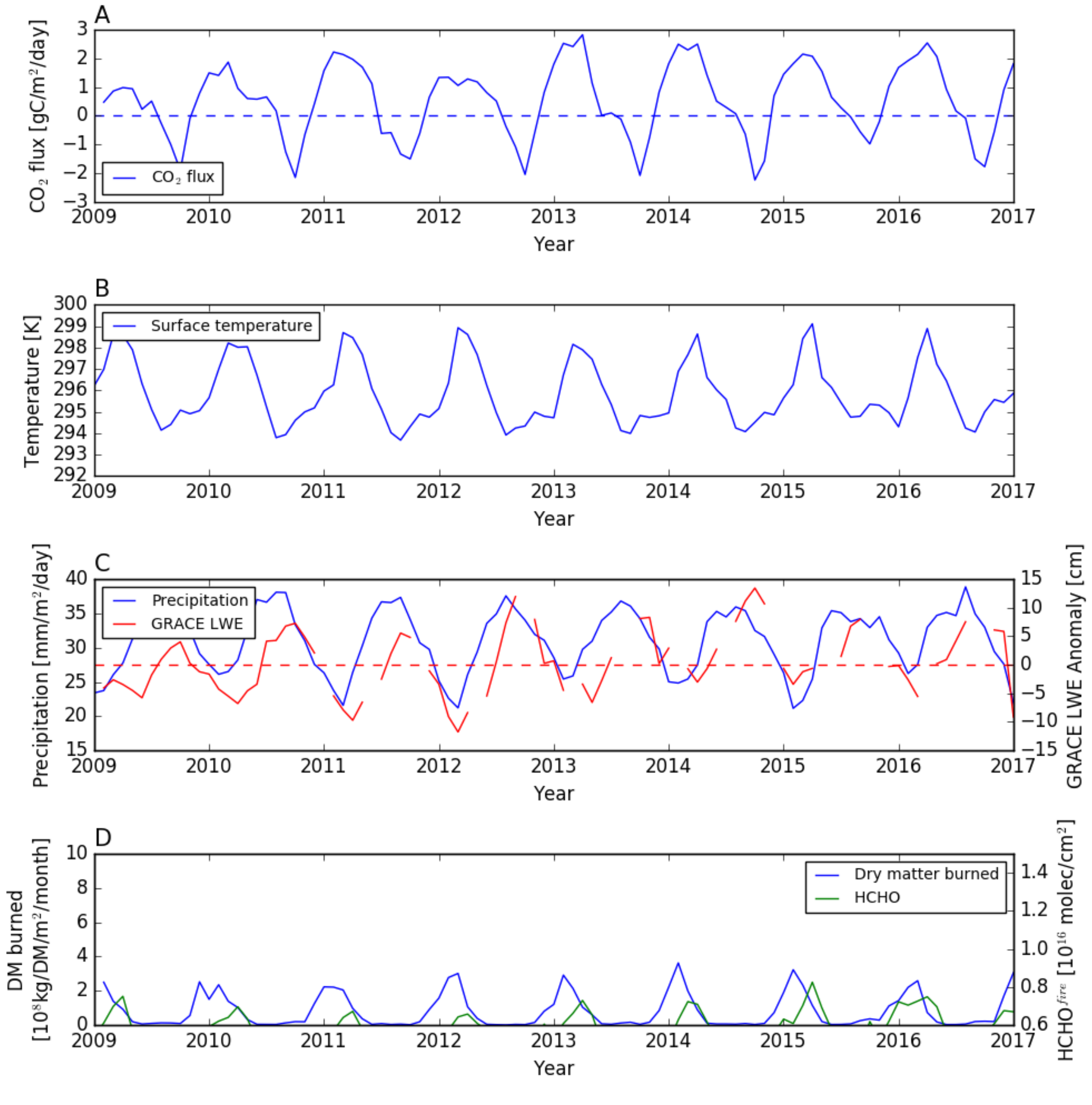

Supplementary Figure 26: Timeseries of A) *a posteriori* CO_2 flux [gC/m^2/day], analyzed B) surface temperature (K) and C) precipitation [mm/m^2/day], and D) dry matter (DM) burned [10^8kg/DM/m^2/month] for western Ethiopia (4–14°N, 33–38°E).

Supplementary Tables

Geographical region	2015						2016					
	**A priori**	**A posteriori**					**A priori**	**A posteriori**				
		**GOSAT**		**OCO-2**				**GOSAT**		**OCO-2**		
		UoE ACOS	UoE UoL	UoE	LSCE	CSU		UoE ACOS	UoE UoL	UoE	LSCE	CSU
Land regions												
North American Boreal	0.14	0.58	-0.27	-0.15	-0.00	-0.27	-0.05	–	-0.83	-0.35	-0.01	-0.62
North American Temperate	-0.03	-0.59	-1.35	-0.75	-1.11	-0.75	-0.13	–	-1.08	-0.55	-1.13	-0.57
Northern Tropical South America	-0.08	-0.27	0.17	0.22	0.23	-0.05	0.00	–	0.17	0.16	0.39	-0.06
Southern Tropical South America	-0.01	-0.31	-0.50	-0.31	-0.19	-0.21	0.35	–	-0.06	0.12	0.14	-0.15
South American Temperate	0.27	-0.15	-0.53	-1.04	-0.90	-0.88	0.33	–	-0.16	-1.11	-0.79	-1.22
North extratropical Africa	-0.01	0.04	0.02	0.03	0.02	-0.05	0.01	–	0.09	0.15	0.07	-0.03
Northern Tropical Africa	-0.05	0.60	0.69	1.89	1.54	1.91	0.06	–	1.45	2.02	1.34	2.54
Southern Tropical Africa	-0.13	0.31	0.11	-0.36	-0.06	0.04	-0.16	–	0.05	-0.23	-0.20	-0.12
South extratropical Africa	-0.05	0.04	0.02	0.08	0.07	-0.01	-0.03	–	-0.02	0.03	0.04	-0.08
Eurasia Boreal	-0.10	-0.16	0.05	-0.16	-0.01	-0.41	0.19	–	0.05	-0.37	-0.36	-0.85
Eurasia Temperate	-0.09	-0.85	0.07	-0.87	-0.47	-0.50	0.03	–	-0.34	0.00	-0.25	0.30
Northern Tropical Asia	0.04	-0.45	0.19	-0.53	-0.22	-0.32	0.16	–	0.18	-0.54	-0.12	-0.06
Southern Tropical Asia	0.18	-0.00	0.21	0.26	0.09	0.42	0.03	–	0.11	0.14	0.10	0.10
Tropical Australia	-0.02	-0.08	-0.33	-0.14	-0.10	-0.06	0.01	–	-0.51	-0.08	-0.05	-0.14
Temperate Australia	-0.03	-0.12	-1.20	0.29	0.17	-0.14	0.00	–	-1.25	-0.45	-0.09	-0.47
Europe	0.01	-0.29	-0.73	-0.09	-0.49	-0.90	-0.07	–	-0.68	-0.37	-0.24	-1.00
Ocean regions												
North Pacific Temperate	-0.48	-0.65	-0.47	-0.54	-1.02	-0.66	-0.48	–	-0.47	-0.52	-0.99	-0.66
West Pacific Tropical	0.04	-0.07	0.05	0.03	-0.09	0.04	0.04	–	0.04	-0.00	-0.34	0.03
East Pacific Tropical	0.40	0.12	0.41	0.35	-0.00	0.44	0.40	–	0.42	0.35	-0.09	0.42
South Pacific Temperate	-0.26	-0.31	-0.28	-0.25	-0.16	-0.30	-0.26	–	-0.28	-0.27	-0.51	-0.30
Northern Ocean	-0.26	-0.35	-0.32	-0.33	-0.27	-0.30	-0.26	–	-0.31	-0.31	-0.25	-0.32
North Atlantic Temperate	-0.23	-0.37	-0.24	-0.24	-0.44	-0.21	-0.23	–	-0.22	-0.20	-0.11	-0.22
Atlantic Tropical	0.11	0.03	0.08	0.13	0.32	0.12	0.11	–	0.12	0.12	0.18	0.12
South Atlantic Temperate	-0.09	-0.13	-0.15	-0.07	-0.13	-0.13	-0.09	–	-0.13	-0.12	-0.29	-0.15
Southern Ocean	-0.43	-0.52	-0.49	-0.40	-0.16	-0.29	-0.43	–	-0.48	-0.45	-0.43	-0.34
Indian Tropical	0.12	0.25	0.16	0.10	-0.12	0.10	0.12	–	0.13	0.11	0.16	0.11
South Indian Temperate	-0.34	-0.27	-0.39	-0.33	-0.15	-0.40	-0.34	–	-0.39	-0.36	-0.32	-0.40

Table 1: *A priori* and *a posteriori* net CO_2 flux estimates (PgC/yr) for 2015 and 2016 for individual geographical regions, including those shown in Table 1 from the main paper, inferred from GOSAT and OCO-2 X_{CO2} retrievals. A hypthen denotes absence of data. UoL and ACOS denote independent retrieval data products from the University of Leicester and the NASA Atmospheric CO_2 Observation from Space, respectively. UoE, LSCE, and CSU denote the modelling groups from the University of Edinburgh, Laboratoire des sciences du climat et de l′environnement, and Colorado State University, respectively.

Supplementary Note 1

A posteriori regional CO_2 fluxes

Figures 1 to 6 show monthly *a posteriori* CO_2 net fluxes expressed as PgC/yr for TransCom geographical regions [15]. These figures complement those shown in the main paper. Table 1 summarizes the annual CO_2 flux estimates inferred from GOSAT (using GEOS-Chem) and OCO-2 (all three transport and inverse method configurations) as described in the methods sections of the main paper.

For much of the world, *a posteriori* CO_2 fluxes inferred from GOSAT and OCO-2 track each other during the 2015–2016 timeframe of the main study. At high latitudes (Figures 1 and 4), there is better agreement between *a posteriori* flux estimates during summer months when there are more data available; during winter months flux estimates are determined by lower latitude regions and by *a priori* estimates. This helps to explain variations in the net annual fluxes for these regions (Table 1).

At temperate latitudes, we find generally larger annual mean sinks, e.g. over North America and South America. All analyses of GOSAT and OCO-2 X_{CO2} are consistent with a large land sink over temperate North America with OCO-2 data associated with a sink larger than GOSAT data. X_{CO2} data are consistent with temperate South America being a large sink in 2015 and 2016.

Ocean flux estimates typically do not deviate far from *a priori* values. This reflects tighter constraints on ocean flux estimates and also that observed variations of X_{CO2} are more likely to be determined by continental outflow than diffuse fluxes from the ocean.

Using the GEOS-Chem model we produced a self-consistent record of *a posteriori* CO_2 flux estimates inferred from two independently retrieved GOSAT X_{CO2} data products (2009–present) to explore how representative the 2015–2016 period is to past years. Figure 7 reports a graphical summary of annual *a priori* and *a posteriori* net CO_2 flux estimates 2010–2015 inferred from GOSAT X_{CO2} over tropical geographical regions. Availability of the ACOS products defined the later time period. Northern tropical South America is in net carbon balance, while southern tropical South America is a small sink that is close to net carbon balance. The two GOSAT data products consistently produce a large net carbon source over northern tropical Africa, albeit smaller than during 2015/2016. However, they diverge over southern tropical Africa, where the UoL data are consistent with a large sink while the ACOS product is close to carbon balance. GOSAT data products over tropical Asia are consistent with net carbon balance. GOSAT data products diverge over Tropical Australia with UoL data consistent with a net sink while the ACOS product is in net balance, close to the *a priori* flux estimates.

Supplementary Note 2

Spatial distribution of CO_2 fluxes over tropical continents

We show the spatial distributions of CO_2 fluxes, corresponding to the line plots shown by Figures 1 to 6, with the caveat that caution should be exercised when interpreting geographical regions $<$ 1000 km in length.

Figures 8 and 9 shows the annual distribution of *a posteriori* CO_2 fluxes over the tropics, incorporating all of our tropical study regions, from 2010 to 2016 for GOSAT and from 2015 to

2016 for OCO-2. For GOSAT, these distributions correspond to values shown in Figure 7 and Table 1. For all plots we have hatched regions where the absolute value of the *a posteriori* flux estimate is larger than 1.25 times its corresponding uncertainty. We include the 25% factor as an acknowledgement that *a posteriori* flux uncertainties are typically optimistic.

The two main observations from these figures are 1) both GOSAT and OCO-2 data infer very similar distributions of *a posteriori* CO_2 fluxes during their period of temporal overlap; and 2) peak annual uptake regions are consistently over southern tropical South America and over the Congo basin, as expected given the distribution of vegetation, and the peak annual emissions vary with time but in recent years are located over semiarid western Ethiopia, western tropical Africa, and Mozambique.

Figures 10 and 11 show seasonal distributions of CO_2 fluxes inferred from OCO-2 during 2015. These complement Figures 1 to 6 and Figure 9. We are only beginning to understand the size of the Congo basin net carbon sink [12, 31, 10], but the distribution and diversity of ecosystems over this region is consistent with the size of the carbon sink we infer from OCO-2 and GOSAT data. The large-scale seasonal CO_2 flux distributions inferred from OCO-2 are similar to GOSAT and are not shown. The distribution of net emissions is consistent with land-use change estimates inferred from microwave data, e.g. [7]. Recent work using dynamical global vegetation models has suggested that historical emissions of CO_2 from land-use change has been underestimated [2], implying that, based on global mass balance, uptake has also been underestimated. This is at least qualitatively consistent with our paper. In the main paper we suggest that soil respiration could play a role in emissions particularly over western Ethiopia [20, 4]. Extensive conversion from natural ecosystems to agricultural land has been estimated to have resulted in a 20–50% reduction of soil carbon stocks [4], suggesting large emissions, but these estimates have large uncertainties.

In an effort to test the robustness of this result over tropical North Africa, we evaluated our *a posteriori* CO_2 fluxes using aircraft data from the Comprehensive Observation Network for TRace gases by AIrLiner (CONTRAIL, [21]). Figure 12 shows that CO_2 fluxes inferred from OCO-2 using GEOS-Chem are more consistent with CONTRAIL data than fluxes inferred from ground-based data between 2015–2017. We have also inferred CO_2 fluxes from these sparse data (not shown). These data significantly increase emission estimates from tropical North Africa, particularly after 2013 when data were collected over the Indian sub-continent and over the western part of tropical Africa (Figure 12). Compared to OCO-2 and GOSAT, CONTRAIL data places the elevated emissions over the northern part of the Congo basin. These differences are likely due to the sparse coverage of CONTRAIL data and the increased transport model errors associated with interpreting these upper tropospheric data.

Supplementary Note 3

Sensitivity of Tropical North African CO_2 flux to potential sources of X_{CO2} retrieval systematic error

The unexpected large tropical north African CO_2 seasonal cycle and annual mean net flux are a robust feature inferred from GOSAT and OCO-2 data using a range of atmospheric transport models and inverse methods. Our findings are also qualitatively consistent with a range of correlative land-surface data and quantitatively consistent with the sparse atmospheric data available to independently evaluate our results.

While there is no evidence to suggest that OCO-2 and GOSAT data have consistent

systematic bias that affects only northern tropical Africa during a particular season, we have used a series of numerical experiments to understand how contrived patterns of systematic error would affect inferred CO_2 fluxes and the size of bias necessary to remove the net emission.

First, we investigate whether the persistent bright surfaces over the Sahara, which can result in erroneously low X_{CO2} retrievals, translate into erroneously large CO_2 flux estimates over northern tropical Africa.

Using the GEOS-Chem model and the same model/inverse method configuration used throughout this study, we discard any observations over the Sahara desert, defined here as 15°W–25°E and 16°–25°N, and calculate the corresponding CO_2 flux estimates. These typically represent 35% of all observations per month over North Africa throughout the year. Figure 13 shows that removing measurements over the Sahara makes little difference to the size of the seasonal cycle or the net flux. We find no evidence of seasonal dust transport over western Ethiopia that would directly affect X_{CO2} retrievals during MAM.

Second, we use a closed-loop experiment that allows us to define the true surface fluxes and the corresponding X_{CO2} distribution following OCO-2 data. We use ORCHIDEE CO_2 fluxes [18] to determine the true X_{CO2} distribution. We use CASA CO_2 fluxes to determine our *a priori* X_{CO2} distribution to which we add a random error (1σ of 1 ppm). Figure 14 shows the results from three inversions: a control calculation (as described above) and two inversions that correspond to a uniform systematic error of ±1 ppm imposed on the true X_{CO2} distribution over northern tropical Africa (15°W–50°E and 0°–32°N). This is a contrived scenario but it serves to illustrate the difficulty in reducing our flux estimates over northern tropical Africa. Figure 14 shows that the control inversion reproduces the truth, as expected. Including systematic error does not significantly affect the amplitude of the seasonal cycle but increases/decreases net annual mean fluxes. The inversion that includes observations with a +1 ppm systematic error results in an annual budget that is 1.35 PgC larger than the control calculation. Using a -1 ppm systematic error results in an annual budget that is 1.47 PgC smaller than the control calculation. With this brute force approach we would need a uniform systematic X_{CO2} error 1.36–1.48 ppm to remove the significant annual mean net emissions of 2 PgC.

Figure 14 also shows that data with systematic errors of ±1 ppm do not significant influence *a posteriori* fluxes over Southern tropical South America. This results supports that OCO-2 and GOSAT X_{CO2} can retrieve independent fluxes for tropical South America and tropical North Africa.

Finally, we determine using an *ad hoc* approach the size of systematic error necessary to remove net emissions over tropical north Africa if they were localized closer to the emission foci (10°W–50°E and 0°–15°N). We find that a systematic error of 2.3 ppm is necessary to achieve our goal. We anticipate that data with systematic errors of that magnitude would be identified by other data.

Sensitivity of Tropical North African CO_2 flux to potential seasonal sampling bias

Figure 15 shows the global clear-sky OCO-2 data coverage for contrasting months in the seasonal of CO_2 fluxes over northern Tropical Africa. Gaps are generally due to clouds and aerosols. The values of X_{CO2} are generally higher during March, as reflected in the inferred fluxes, and there are a comparable number of observation available for both March and September over northern tropical Africa. Caution should be exercised when interpreting

spatial patterns of X_{CO2}. Local variations of X_{CO2} do not directly equate to local changes in CO_2 fluxes. A CO_2 column represents a weighted atmospheric column with contributions from throughout the troposphere originating from many different geographical regions and time periods, with local surface emissions and uptake representing only a small fraction of the column amount. Consequently, a 3-D model of atmospheric transport is needed to interpret the columns.

A temporal sampling bias is unlikely because there are clouds over many of these key regions throughout the year, with biomass burning aerosols also playing a role in data removal. A spatial sampling bias is possible with a large number of measurements over less cloudy regions. However, a regional flux is not estimated exclusively using data immediately overhead. There remains substantial information in columns observed at later times downwind of a cloudy region [14]. This is true elsewhere in the tropics.

To illustrate this point we have included an additional calculation, based on the calculations shown in the main paper and SI, to evaluate the effectiveness of OCO-2 measurements to estimate a monthly flux estimate. As an example we focus on a geographical region over tropical Africa (Figure 16, left panel) and determine the error reduction associated with a monthly flux estimate at time = 0 due to data collected on later days. We report calculations for two contrasting months: September 2015 and March 2016. We take into account model transport error.

In the Kalman Filter framework [14], the *a posteriori* flux error covariance ($\mathbf{P}^a$) is determined by:

$$\mathbf{P}^a = (1 - \mathbf{P}^f\mathbf{H}^T[\mathbf{R} + \mathbf{P}^f\mathbf{H}\mathbf{H}^T]^{-1}\mathbf{H})\mathbf{P}^f, \tag{1}$$

where $\mathbf{P}^f$ represents the *a priori* error covariance, $\mathbf{H}$ is the Jacobian matrix that relates surface fluxes from the target region to atmospheric measurements (X_{CO2}). We use the GEOS-Chem model to numerically calculate the Jacobian matrix by perturbing the a priori emission [14] and to the solve the equation using a sequential approach. Figure 16 (right panel) shows the error reduction metric $\gamma = 1 - \sqrt{\mathbf{P}^a_i/\mathbf{P}^f_i}$, where $\mathbf{P}^a_i$ and $\mathbf{P}^f_i$ denote the *a posteriori* and *a priori* error covariance matrices determined by sequential assimilation approach from day 1 to day i. Our calculation shows that gamma only reaches 0.2 within a few days and reaches 0.5 at 20 days and begins to converge to the final value of 0.8 at 40+ days. This shows that our knowledge of regional CO2 fluxes is not determined exclusively from data collected immediately over the study region at one time. Small differences between the error reductions from September 2015 and March 2016 reflect differences in the distribution of available data, and confirm that fluxes from these contrasting months (characterizing the peaks of the seasonal cycle over this geographical region) are determined by observations.

Supplementary Note 4

Independent correlative data for geographical regions

Figures 17 to 22 show correlative data used to interpret temporal and spatial distributions of CO_2 flux estimates inferred from OCO-2 and GOSAT observations of X_{CO2}. We have shown these figures from 2009–2016, inclusive, for completeness sake. Data are described in the methods section of the main paper.

Figure 23 shows a summary of monthly SIF values over key tropical regions. SIF values over northern tropical Africa are smaller than values found over other tropical regions and

with a muted seasonal cycle. This suggests that SIF, which is closely related to GPP [26], is not responsible for the large seasonal cycle over northern Tropical Africa.

Supplementary Discussion

Carbon sources over northern Tropical Africa and western Ethiopia

Figure 3 shows our headline result of a larger than expected seasonal cycle of carbon fluxes over northern tropical Africa, resulting in a net annual mean flux to the atmosphere. Figures 8–10 show that these net annual mean fluxes to the atmosphere result from a band of elevated annual net emissions with foci over western Ethiopia and western tropical Africa. Here, we discuss our headline result in the context of the ORCHIDEE land surface model that was used by the LSCE group (Figures 1 and 2 from the main paper and Figures 1 to 6 from the SI). The ORCHIDEE model generally performs well against independent correlative data [19]. We then discuss in more detail possible carbon sources over western Ethiopia.

Carbon sources over northern Tropical Africa

Figure 24 shows that the ORCHIDEE land surface model describes the magnitude and approximate timing (peaking a month earlier) of the seasonal cycle over northern Tropical Africa inferred from OCO-2 and GOSAT X_{CO2} data. Gross primary production (GPP) has a large seasonal cycle that is weakest (smallest loss of carbon from the atmosphere) during February and March. Heterotrophic respiration is relatively constant throughout the year, while autotrophic respiration has a shallow seasonal cycle that is anti-correlated to GPP. This is reinforced by a small fire flux that peaks during February and March over the broad geographical region. The result is the net biospheric flux peaks in February and reduces rapidly with GPP. Both observed and model net fluxes have net uptake later in the year, with a larger model uptake during July and August that results in an approximate annual carbon balance.

A significant net annual flux to the atmosphere (i.e. net emission) is possible as part of year to year variations with decreasing (increasing) GPP (respiration terms or fire) but this is generally considered unsustainable since we have to assume carbon is conserved on longer times: carbon produced during photosynthesis is later lost (mostly) through respiration and fires. However, if there exists a vulnerable carbon store that is being continually depleted, that would effectively shift upwards the net biospheric flux, resulting in an annual net emission that peaks in March and April. The timing of the peak in ORCHIDEE is determined mostly by GPP (Figure 24). A carbon store that is vulnerable to warmer temperatures would exacerberate the situation by partly offsetting the seasonal cycle of GPP, and effectively further weaken the period of net uptake.

The alternative hypothesis is more speculative, developed by deduction using available data: an additional, unknown seasonal emission of carbon that peaks in spring months. The large net emission of CO_2 focused over western Ethiopia peaks broadly in the MAM season when the surface environment is hottest and driest (Figure 25). Values found over western Ethiopia during MAM are typically not found over other tropical environments, e.g. Amazon basin. That observation appears to be consistent with a seasonal source, but as we argue above a seasonal peak could result from a larger than expected aseasonal souce. Here, we

provide an assessment of known fluxes and put forward a speculative seasonal source. In practice, we do not preclude the possibility that our signal is some combination of sources.

Fire associated with agricultural practices follows the same seasonality as the observed net CO_2 fluxes.Elevated space-borne observations of atmospheric tracers of incomplete combustion, e.g. carbon monoxide from the Measurement of Pollution In The Troposphere (not shown) and formaldehyde from the Ozone Monitoring Instrument (main text and Figure 26), and of data products derived from land surface properties, e.g. dry matter burned (main text) cannot explain the observed carbon emission. Recent work has highlighted that burning over this region is also a major source of ammonia [29].

Carbon sources over western Ethiopia

Western Ethiopia has some of the highest values of soil organic carbon anywhere in the world, in excess of 150 Mg/hectare [13]. We cannot discount a role for elevated soil respiration associated with higher temperature, but previous studies have emphasized the importance of a soil moisture with respiration following a bell curve based on soil moisture (e.g. [32]). Based on satellite data and analyzed meteorology (Methods) we find that over western Ethiopia monthly CO_2 fluxes increase when temperature rises, and when precipitation and liquid water equivalent thickness falls (Figure 26). Western Ethiopia has also experienced extensive and persistent land use change [5] with significant reductions in above-ground biomass [3, 7] and soil erosion (e.g. [27]). Soil degradation due to land-use change could exacerbate carbon loss but empirical evidence is inconclusive [30, 11, 6]. Continued land degradation and warming temperatures could potentially result in a large diffuse CO_2 emission source with a temperature dependence. We acknowledge the influence of climate warming on net carbon emissions from soil is non-trivial and subject to a number of confounding factors [22].

There are also arid and semi-arid ecosystems that neighbour western Ethiopia. Previous studies have highlighted a possible role for CO_2 fluxes from biological soil crusts that contain myriad biological communities (e.g., cyanobacteria, lichens). These crusts can form material that is resistance to wind and water erosion [24]. Mesocosm experiments appear to show biocrusts can be a CO_2 source during hot summer months but are linked with pulsed wetting events [28]. It is unlikely they will play a major role (if any) in explaining the large source of CO_2 over western Ethiopia.

The east African rift is a dominant geological feature of Ethiopia (e.g. [16]). Deep carbon emissions have been the subject of a number of studies (e.g. [23, 8], including diffuse volcanic degassing, geothermal lakes and wells, faults, and fumaroles. These emissions have been the subject to a number of studies that conclude emissions ranging between 4–104 $TgCO_2$/yr ([16] and references therein). While they undoubtedly will play a role in CO_2 emission over Ethiopia, they will do not explain observations: they tend to be further east of where we observe our largest emissions, there is no underlying reason why these emissions would peak in MAM, and bottom-up estimates are an order of magnitude too small to explain fluxes inferred from GOSAT and OCO-2.

A more speculative seasonal source over western Ethiopia, developed by linking previously published data, is from inorganic carbon emissions that originate from karsts. Karsts are caves formed from the dissolution of soluble rock [9], a process that occurs over thousands of years. Here, we explore how the unique environment over western Ethiopia could in theory result in karst carbonate chemistry producing a CO_2 pulse at the beginning of the dry season. Over Ethiopia more than 20% of the land includes lithology that is karstifiable, e.g. limestone,

dolomite, marble, and sandstone [17], which are mainly located in the Mekele region, the northeastern plateau close to Somalia, the Blue Nile gorge, and the Afar depression. Western Ethiopia also has high values of soil organic carbon stores that are subject to precipitation and high temperatures. We propose that during the rainy season, soil water originating from precipitation results in large amounts of weak carbonic acid that eventually precipitates in the underlying karst structures, building on [25] (and references therein). The carbonic acid converts calcite into calcium bicarbonate when it seeps through joints and fissures of a karst region: $CO_2 + H_2O + CaCO_3 \rightarrow Ca(HCO_3)_2$. When the water eventually reaches the roof of the karst structure it forms a droplet that eventually drops to the floor. The water droplet will contain a higher concentration of dissolved CO_2 than the ambient air in the cave so some CO_2 will be released to the air. To re-establish chemical equilibrium a small amount of the $CaCO_3$ is deposited onto the cave roof. In a closed karst system the only way to vent this inorganic atmospheric CO_2 is from above. During the wet season, soils are wet and can significantly impede the diffusion of gas to the overlying atmosphere [1]. Approaching the dry season, as the soil becomes drier, the inorganic CO_2 that slowly built up during the wet season can begin to escape to the atmosphere, resulting in a pulse that peaks in MAM. This assumes a vertical atmospheric temperature gradient that promotes the rising karst air through the soil membrane towards the atmosphere. We acknowledge the magnitude of this pulse depends on a number of factors, including, for example, a large karst cavity capacity, rainfall amounts, and the biological productivity of the soils. We emphasize this is a speculative hypothesis that builds on a range of published data.

Supplementary References

[1] S. E. Allaire, J. A. Lafond, A. R. Cabral, and S. F. Lange. Measurement of gas diffusion through soils: comparison of laboratory methods. *J. Environ. Monit.*, 10:1326–1336, 2008.

[2] A. Arneth, S. Sitch, J. Pongratz, B. D. Stocker, P. Ciais, B. Poulter, A. D. Bayer, A. Bondeau, L. Calle, L. P. Chini, T. Gasser, M. Fader, P. Friedlingstein, E. Kato, W. Li, M. Lindeskog, J. E. M. S. Nabel, T. A. M. Pugh, E. Robertson, N. Viovy, C. Yue, and S. Zaehle. Historical carbon dioxide emissions caused by land-use changes are possibly larger than assumed. *Nature Geoscience*, 10(2):79+, FEB 2017.

[3] A. Baccini, W. Walker, L. Carvalho, M. Farina, D. Sulla-Menashe, and R. A. Houghton. Tropical forests are a net carbon source based on aboveground measurements of gain and loss. *Science*, 2017. doi:10.1126/science.aam5962.

[4] T. Berihu, G. Girmay, M. Sebhatleab, E. Berhane, A. Zenebe, and G. C. Sigua. Soil carbon and nitrogen losses following deforestation in Ethiopia. *Agronomy for Sustainable Development*, 37(1), FEB 2017.

[5] T. Betru, M. Tolera, K. Sahle, and H. Kassa. Trends and drivers of land use/land cover change in western ethiopia. *Applied Geography*, 104:83 – 93, 2019.

[6] P. Borrelli, D. A. Robinson, L. R. Fleischer, E. Lugato, C. Ballabio, C. Alewell, K. Meusburger, S. Modugno, B. Schütt, V. Ferro, V. Bagarello, K. V. Oost, L. Montanarella, and P. Panagos. An assessment of the global impact of 21st century land use chage on soil erosion. *Nature Communications*, 2017.

[7] M. Brandt, J.-P. Wigneron, J. Chave, T. Tagesson, J. Penuelas, P. Ciais, K. Rasmussen, F. Tian, C. Mbow, A. Al-Yaari, N. Rodriguez-Fernandez, G. Schurgers, W. Zhang, J. Chang, Y. Kerr, A. Verger, C. Tucker, A. Mialon, L. V. Rasmussen, L. Fan, and R. Fensholt. Satellite passive microwaves reveal recent climate-induced carbon losses in African drylands. *Nature Ecology & Evolution*, 2(5):827–835, MAY 2018.

[8] M. R. Burton, G. M. Sawyer, and D. Granieri. Deep carbon emissions from volcanoes. *Reviews in Mineralogy and Geochemistry*, 75(1):323, 2013.

[9] A. S. A. Chen, Z., M. Bakalowicz, D. Drew, F. Griger, J. Hartmann, G. J. ans N. Moosdorf, A. Richts, Z. Stevanovic, G. Veni, and N. Goldscheider. The world karst aquifer mapping project: concept, mapping procedure and map of europe. *Hydrogology Journal*, 2017.

[10] G. C. Dargie, S. L. Lewis, I. T. Lawson, E. T. A. Mitchard, S. E. Page, Y. E. Bocko, and S. A. Ifo. Age, extent and carbon storage of the central Congo Basin peatland complex. *Nature*, 2017. doi:10.1038/nature21048.

[11] S. Doetterl, A. A. Berhe, E. Nadeu, Z. Wang, M. Sommer, and P. Fiener. Erosion, deposition and soil carbon: A review of process-level controls, experimental tools and models to address c cycling in dynamic landscapes. *Earth-Science Reviews*, 2016.

[12] S. Doetterl, E. Kearsley, M. Bauters, K. Hufkens, J. Lisingo, G. Baert, H. Verbeeck, and P. Boeckx. Aboveground vs. Belowground Carbon Stocks in African Tropical Lowland Rainforest: Drivers and Implications. *PLOS One*, 10(11), NOV 24 2015.

[13] FAO. Global soil organic carbon map. Technical report, FAO, 2018.

[14] L. Feng, P. I. Palmer, H. Bösch, and S. Dance. Estimating surface co_2 fluxes from space-borne co_2 dry air mole fraction observations using an ensemble kalman filter. *Atmospheric Chemistry and Physics*, 9(8):2619–2633, 2009.

[15] K. Gurney, R. Law, A. Denning, P. Rayner, D. Baker, P. Bousquet, L. Bruhwiler, Y. Chen, P. Ciais, S. Fan, I. Fung, M. Gloor, M. Heimann, K. Higuchi, J. John, T. Maki, S. Maksyutov, K. Masarie, P. Peylin, M. Prather, B. Pak, J. Randerson, J. Sarmiento, S. Taguchi, T. Takahashi, and C. Yuen. Towards robust regional estimates of CO2 sources and sinks using atmospheric transport models. *NATURE*, 415(6872):626–630, FEB 7 2002.

[16] J. A. Hunt, A. Zafu, T. A. Mather, D. M. Pyle, and P. H. Barry. Spatially variable co2 degassing in the main ethiopian rift: Implications for magma storage, volatile transport, and rift-related emissions. *Geochemistry, Geophysics, Geosystems*, 18(10):3714–3737, 2017.

[17] S. Kebede. *Groundwater in Ethiopia.* Springer-Verlag Berlin Heidelberg, 2013. doi:10.1007/978-3-642-30391-3.

[18] G. Krinner, N. Viovy, N. de Noblet-Ducoudr, J. Oge, J. Polcher, P. Friedlingstein, P. Ciais, S. Sitch, and I. C. Prentice. A dynamic global vegetation model for studies of the coupled atmosphere-biosphere system. *Global Biogeochemical Cycles*, 19(1), 2005.

[19] C. Le Quéré, R. M. Andrew, P. Friedlingstein, S. Sitch, J. Hauck, J. Pongratz, P. A. Pickers, J. I. Korsbakken, G. P. Peters, J. G. Canadell, A. Arneth, V. K. Arora, L. Barbero, A. Bastos, L. Bopp, F. Chevallier, L. P. Chini, P. Ciais, S. C. Doney, T. Gkritzalis, D. S. Goll, I. Harris, V. Haverd, F. M. Hoffman, M. Hoppema, R. A. Houghton, G. Hurtt, T. Ilyina, A. K. Jain, T. Johannessen, C. D. Jones, E. Kato, R. F. Keeling, K. K. Goldewijk, P. Landschützer, N. Lefèvre, S. Lienert, Z. Liu, D. Lombardozzi, N. Metzl, D. R. Munro, J. E. M. S. Nabel, S.-I. Nakaoka, C. Neill, A. Olsen, T. Ono, P. Patra, A. Peregon, W. Peters, P. Peylin, B. Pfeil, D. Pierrot, B. Poulter, G. Rehder, L. Resplandy, E. Robertson, M. Rocher, C. Rödenbeck, U. Schuster, J. Schwinger, R. Séférian, I. Skjelvan, T. Steinhoff, A. Sutton, P. P. Tans, H. Tian, B. Tilbrook, F. N. Tubiello, I. T. van der Laan-Luijkx, G. R. van der Werf, N. Viovy, A. P. Walker, A. J. Wiltshire, R. Wright, S. Zaehle, and B. Zheng. Global carbon budget 2018. *Earth System Science Data*, 10(4):2141–2194, 2018.

[20] M. Lemenih, E. Karltun, and M. Olsson. Soil organic matter dynamics after deforestation along a farm field chronosequence in southern highlands of Ethiopia. *Agriculture Ecosystems & Environment*, 109(1-2):9–19, AUG 1 2005.

[21] H. Matsueda, H. Inoue, and M. Ishii. Aircraft observation of carbon dioxide at 8-13 km altitude over the western Pacific from 1993 to 1999. *Tellus Series B-Chemical and Physical Meteorology*, 54(1):1–21, FEB 2002.

[22] J. M. Melillo, S. D. Frey, K. M. DeAngelis, W. J. Werner, M. J. Bernard, F. P. Bowles, G. Pold, M. A. Knorr, and A. S. Grandy. Long-term pattern and magnitude of soil carbon feedback to the climate system in a warming world. *Science*, 358(6359):101–105, 2017.

[23] N.-A. Mrner and G. Etiope. Carbon degassing from the lithosphere. *Global and Planetary Change*, 33(1):185 – 203, 2002. The global carbon cycle and its changes over glacial-interglacial cycles.

[24] L. G. Sancho, J. Belnap, C. Colesie, J. Raggio, and B. Weber. *Biological soil crusts: an organising principle in drylands*, chapter Carbon budgets of biological soil crusts at micro-, meso-, and global scales. Springer International Publishing, 2016. doi:10.1007/978-3-319-30214-0_15.

[25] P. Serrano-Ortiz, M. Roland, S. Sanchez-Moral, I. A. Janssens, F. Domingo, Y. Goddris, and A. S. Kowalski. Hidden, abiotic co2 flows and gaseous reservoirs in the terrestrial carbon cycle: Review and perspectives. *Agricultural and Forest Meteorology*, 150(3):321 – 329, 2010.

[26] Y. Sun, C. Frankenberg, M. Jung, J. Joiner, L. Guanter, P. Khler, and T. Magney. Overview of solar-induced chlorophyll fluorescence (sif) from the orbiting carbon observatory-2: Retrieval, cross-mission comparison, and global monitoring for gpp. *Remote Sensing of Environment*, 209:808 – 823, 2018.

[27] L. Tadesse, K. Suryabhagavan, G. Sridhar, and G. Legesse. Land use and land cover changes and soil erosion in yezat watershed, north western ethiopia. *International Soil and Water Conservation Research*, 5(2):85 – 94, 2017.

[28] C. L. Tucker, S. Ferrenberg, and S. C. Reed. Climate sensitivity of dryland soil co_2 fluxes differs dramatically with biological soil crust successional state. *Ecosystems*, 2018.

[29] M. Van Damme, L. Clarisse, S. Whitburn, J. Hadji-Lazaro, D. Hurtmans, C. Clerbaux, and P.-F. Coheur. Industrial and agricultural ammonia point sources exposed. *Nature*, 2018.

[30] K. Van Oost, T. A. Quine, G. Govers, S. D. Gryze, J. Six, J. W. Harden, J. C. Ritchie, G. W. McCarty, G. Heckrath, C. Kosmas, J. V. Giraldez, J. R. M. da Silva, and R. Merckx. The impact of agricultural soil erios on the global carbon cycle. *Science*, 2007.

[31] L. Xu, S. S. Saatchi, A. Shapiro, V. Meyer, A. Ferraz, Y. Yang, J.-F. Bastin, N. Banks, P. Boeckx, H. Verbeeck, S. L. Lewis, E. T. Muanza, E. Bongwele, F. Kayembe, D. Mbenza, L. Kalau, F. Mukendi, F. Ilunga, and D. Ebuta. Spatial Distribution of Carbon Stored in Forests of the Democratic Republic of Congo. *Scientific Reports*, 7, NOV 8 2017.

[32] Y. Yohannes, O. Shibistova, A. Abate, M. Fetene, and G. Guggenberge. Soil co_2 efflux in an afromontane forest in ethiopia as driven by seasonality and tree species. *Forest Ecology and Management*, 2011. doi:10.1016/j.foreco.2010.12.032.
