# Supplementary material for: Net carbon emissions from African biosphere dominate pan-tropical atmospheric CO2 signal
Source: Nat Commun. 2019 Aug 13;10:3344. doi: 10.1038/s41467-019-11097-w (PMC6692308; doi:10.1038/s41467-019-11097-w)
Supplement: Supplementary file 2 — Peer Review File [file 41467_2019_11097_MOESM2_ESM.pdf]

REVIEWERS' COMMENTS:

Reviewer #1 (Remarks to the Author):

I have seen the manuscript multiple times by now and think the authors did the best they could at the current stage, this manuscript will trigger stimulating discussions.
